# Mitochondrial IRG1 traps MCL-1 to induce hepatocyte apoptosis and promote carcinogenesis

**DOI:** 10.1038/s41419-023-06155-7

**Published:** 2023-09-22

**Authors:** Liyuan Zhang, Yue Dong, Luxin Zhang, Minjun Wang, Ye Zhou, Kaiwei Jia, Suyuan Wang, Mu Wang, Yunhui Li, Shudan Luo, Shan Lu, Yiwen Fan, Dingji Zhang, Yingyun Yang, Nan Li, Yizhi Yu, Xuetao Cao, Jin Hou

**Affiliations:** 1 National Key Laboratory of Medical Immunology & Institute of Immunology, Second Military Medical University, Shanghai, 200433 China; 2 Department of Cell Biology, Center for Stem Cell and Medicine, Second Military Medical University, Shanghai, 200433 China; 3 Center for Immunotherapy, Chinese Academy of Medical Sciences, Beijing, 100005 China

**Keywords:** Liver cancer, Apoptosis

## Abstract

Hepatocarcinogenesis is initiated by repeated hepatocyte death and liver damage, and the underlying mechanisms mediating cell death and the subsequent carcinogenesis remain to be fully investigated. Immunoresponsive gene 1 (IRG1) and its enzymatic metabolite itaconate are known to suppress inflammation in myeloid cells, and its expression in liver parenchymal hepatocytes is currently determined. However, the potential roles of IRG1 in hepatocarcinogenesis are still unknown. Here, using the diethylnitrosamine (DEN)-induced hepatocarcinogenesis mouse model, we found that IRG1 expression in hepatocytes was markedly induced upon DEN administration. The DEN-induced IRG1 was then determined to promote the intrinsic mitochondrial apoptosis of hepatocytes and liver damage, thus enhancing the subsequent hepatocarcinogenesis. Mechanistically, the mitochondrial IRG1 could associate and trap anti-apoptotic MCL-1 to inhibit the interaction between MCL-1 and pro-apoptotic Bim, thus promoting Bim activation and downstream Bax mitochondrial translocation, and then releasing cytochrome *c* and initiating apoptosis. Thus, the inducible mitochondrial IRG1 promotes hepatocyte apoptosis and the following hepatocarcinogenesis, which provides mechanistic insight and a potential target for preventing liver injury and HCC.

## Introduction

Hepatocellular carcinoma (HCC) is the most prevalent form of primary liver malignancies with increasing incidence and mortality [1, 2]. Infection of hepatitis B or C viruses, metabolic fatty liver disease, aflatoxin B1 exposure, and excessive alcohol intake are the main risk factors for HCC carcinogenesis [3]. The pathophysiology of HCC is made up of multistep processes and accumulated evidences support the inextricably linkage between cell death and hepatocarcinogenesis [4, 5]. In healthy liver, the majority of hepatocytes are in the resting phase without obvious cell death [6, 7]. This resting state could be disrupted by viral, metabolic, toxic or immune injuries, which induce the following death of hepatocytes [4]. Repeated hepatocyte death gives rise to the compensatory proliferation of remaining hepatocytes, which may eventually initiate hepatocarcinogenesis [8]. Thus, excessive hepatocyte death is deemed as a critical driver of acute and chronic liver diseases and a significant determinant of disease outcomes. Therefore, the mechanisms responsible for cell death, hepatic damage, and the subsequent hepatocarcinogenesis require further and intensive investigation.

Different forms of cell death, such as apoptosis, necroptosis, ferroptosis and pyroptosis, are involved with different types of liver cancer substantially [9, 10]. The microenvironment surrounded by apoptotic hepatocytes generates HCC, while cholangiocarcinoma is initiated by necroptosis [11]. For apoptosis, it is induced by extrinsic pathway mediated by death domain-containing adaptors, or intrinsic pathway, which is initiated in the mitochondrion and continued by the release of cytochrome *c* and the activated caspases [12]. The B cell lymphoma-2 (Bcl-2) family members play pivotal roles in the control of mitochondrial intrinsic apoptosis, such as pro-survival proteins MCL-1 and Bcl-2, BH3-only pro-apoptotic proteins Bim, Puma, and Bid, and pro-apoptotic effector proteins Bax and Bak. When the intrinsic apoptotic pathway is activated, the anti-apoptotic proteins interact with the pro-apoptotic ones to determine the fate of the cells [13, 14]. Some of the Bcl-2 family members have been determined to regulate hepatocarcinogenesis by influencing hepatocyte apoptosis. Hepatocyte-specific deletion of pro-survival MCL-1 resulted in augmented hepatic apoptosis and compensatory proliferation, leading to the spontaneous HCC in mice [15]. On the contrary, knockout of pro-apoptotic Puma, Bok, or Bid presented suppressed hepatocarcinogenesis through reducing hepatic apoptosis [16–18]. These evidences corroborate the crucial role of hepatocyte apoptosis in the progression of hepatocarcinogenesis. These works also suggest the significance and concentrated interest in the investigation of the regulators and mechanisms in hepatocyte apoptosis and the subsequent HCC initiation.

The immune-responsive gene 1 (IRG1), also named aconitate decarboxylase 1, is a gene encoding cis-aconitate decarboxylase, a mitochondrial enzyme catalyzing cis-aconitate to itaconate [19, 20]. The roles of IRG1 and its metabolic product itaconate in myeloid cells have been intensively investigated. The prominently upregulated IRG1 induced by pathogen-associated molecular patterns and damage-associated molecular patterns in myeloid cells rendered generation of itaconate to limit inflammation by inhibiting succinate dehydrogenase (SDH) activity or alkylating cysteine residues of multiple proteins [19, 21]. Meanwhile, we also found the upregulated IRG1 in macrophages during sepsis, which increased A20 expression through ROS to strengthen endotoxin tolerance [22]. A recent study of IRG1 in liver macrophages presented that its expression and the product itaconate were decreased during obesity, causing the increased SDH and fumarate hydratase to decrease their substrates succinate and fumarate, thus exaggerating oxidative stress through suppressing NRF2 signals. In liver parenchymal hepatocytes, IRG1 and itaconate were identified to be upregulated to inhibit ischemia-reperfusion injury by activating NRF2 antioxidative response [23, 24]. However, during cell death, hepatic damage, and the subsequent hepatocarcinogenesis, it remains unknown whether and how hepatic IRG1 participates in these disease processes. Moreover, it also needs further investigation that whether the biological functions of IRG1 dependent only on its product itaconate.

Thereout, in order to investigate the underlying roles of IRG1 in hepatocarcinogenesis, we used the chemical carcinogen diethylnitrosamine (DEN)-induced HCC mouse model, and found that DEN administration significantly enhanced IRG1 expression in hepatocytes, and knockout of hepatic IRG1 markedly suppressed DEN-induced HCC, suggesting the potential tumor-promotive roles of IRG1 in hepatocarcinogenesis. Thus, we focused on the roles and underlying mechanisms of hepatic IRG1 in HCC carcinogenesis in this study, so as to provide mechanistic insight and potential target for preventing HCC.

## Results

### DEN-inducible IRG1 in hepatocytes promotes hepatocarcinogenesis

**Fig. 1:**
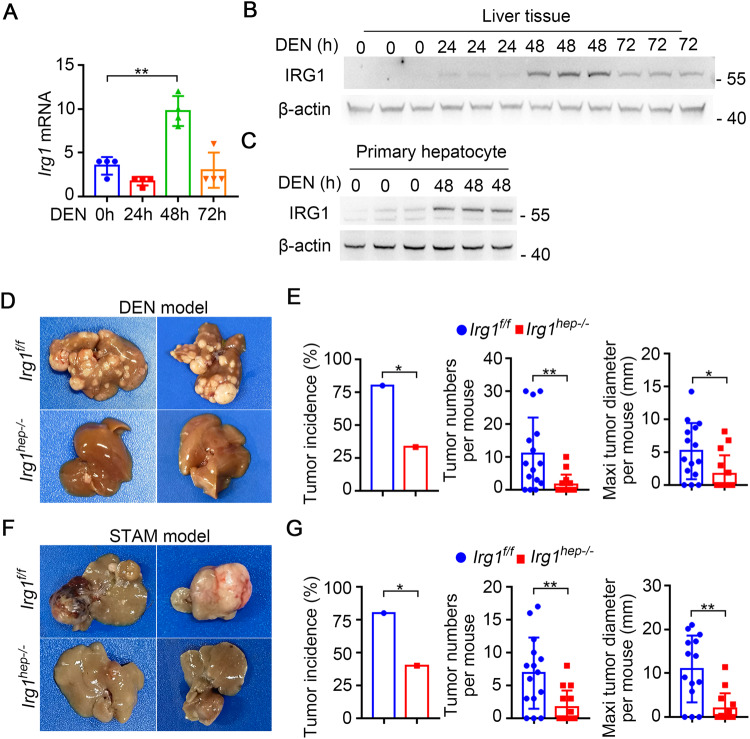
DEN-inducible IRG1 in hepatocytes promotes hepatocarcinogenesis. **A**, **B** *Irg1* expression was examined by qRT-PCR (**A**) and western blot (**B**) in the liver tissues from mice injected with DEN for the indicated time points (*n* = 4, one-way ANOVA). **C** DEN-induced IRG1 expression was examined by western blot in the isolated hepatocytes from the mice treated as in (**B**). **D** Representative livers of DEN-induced HCC in *Irg1*^*f/f*^ and *Irg1*^*hep-/-*^ mice. **E** Tumor incidence (*χ*^2^ test), number and maximum diameter (unpaired *t*-test) in (**D**) were analyzed (*n* = 15). **F** Representative livers of STAM HCC in *Irg1*^*f/f*^ and *Irg1*^*hep-/-*^ mice. **G** Tumor incidence (*χ*^2^ test), number and maximum diameter (unpaired *t*-test) in (**F**) were analyzed (*n* = 15). Data are shown as mean ± SD or photographs from one representative of three independent experiments. *P < 0.05, **P < 0.01.

In order to investigate the potential role of IRG1 in hepatocarcinogenesis, we used the DEN-induced HCC mouse model and examined IRG1 expression upon chemical carcinogen DEN administration. DEN significantly enhanced IRG1 expression at both mRNA and protein levels in the liver, which reached the peak at about 48 h post injection, suggesting the DEN-inducible IRG1 in the liver damage phase (Fig. 1A, B). The primary hepatocytes were isolated, and DEN-induced IRG1 expression was determined in parenchymal hepatocytes (Fig. 1C). In addition, using hepatotoxicant acetaminophen (APAP), metabolized by hepatic cytochrome P450 system as DEN to cause liver damage [25], we found that hepatic IRG1 expression was also significantly enhanced during liver injury (Supplementary Fig. 1A–C). Thus, these data determine the enhanced hepatic IRG1 expression during DEN-induced liver injury, and imply the potential role of IRG1 in hepatocarcinogenesis.

We then constructed hepatocyte-specific IRG1 knockout (*Irg1*^*hep-/-*^) mice, and confirmed the depleted IRG1 expression in the isolated primary hepatocytes (Supplementary Fig. 1D, E). Using DEN-induced hepatocarcinogenesis mouse model, we found that the DEN-induced HCC was markedly suppressed by hepatocyte-specific IRG1 deficiency (Fig. 1D, E). Furthermore, using STAM hepatocarcinogenesis model with streptozotocin and high-fat diet, which was the most molecularly similar to human HCC among the available hepatocarcinogenesis mouse models [26], the induced HCC was also markedly inhibited in *Irg1*^*hep-/-*^ mice (Fig. 1F, G), and hepatic *Irg1* expression was increased during STAM induction process (Supplementary Fig. 1F). Together with the enhanced IRG1 during liver injury, we determined that the inducible hepatic IRG1 expression promoted hepatocarcinogenesis.

### The inducible IRG1 promotes hepatocyte apoptosis and liver injury

**Fig. 2:**
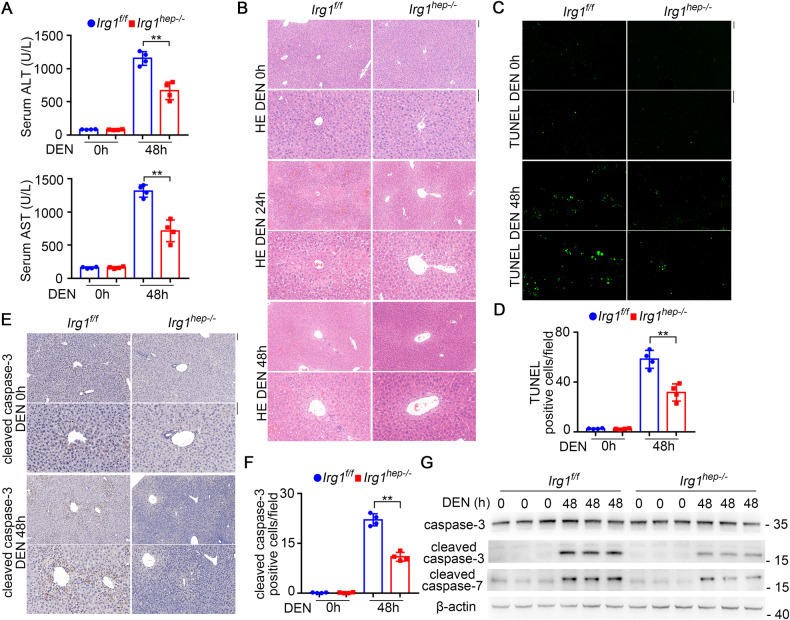
IRG1 promotes hepatocyte apoptosis and liver injury upon DEN administration. **A**–**G** Eight-week-old male *Irg1*^*f/f*^ and *Irg1*^*hep-/-*^ mice were injected with DEN for the indicated time periods. DEN-induced liver damage was measured by serum ALT and AST (**A**, unpaired *t*-test). Liver pathology was analyzed by HE staining (**B**). Hepatic apoptosis was analyzed by TUNEL (**C**, **D**) and cleaved caspase-3 (**E**, **F**) staining (unpaired *t*-test), and western blot (**G**). Scale bars: 100 μm. Data are shown as mean ± SD (*n* = 4) or photographs from one representative of three independent experiments. **P < 0.01.

The underlying mechanism responsible for *Irg1*^*hep-/-*^-mediated inhibition of hepatocarcinogenesis was then investigated. As hepatic IRG1 expression was enhanced upon DEN administration, we examined the DEN-induced liver injury, inflammation, and hepatocyte compensatory proliferation in *Irg1*^*hep-/-*^ mice. Notably, DEN-induced hepatic damage was significantly suppressed in *Irg1*^*hep-/-*^ mice, shown by decreased serum ALT and AST, and by reduced pathological injury in peri-central region of lobules (Fig. 2A, B). Meanwhile, the infiltration of inflammatory cells and compensatory proliferation of hepatocytes were less influenced by hepatic IRG1 deficiency (Supplementary Fig. 2A–H). Furthermore, the DEN-induced apoptosis of hepatocytes was then examined, and we found that hepatocyte apoptosis was significantly inhibited in *Irg1*^*hep-/-*^ livers, shown by the reduced TUNEL staining and caspase-3/7 cleavage (Fig. 2C–G). Thus, DEN-induced IRG1 promotes apoptosis of hepatocytes to worsen liver injury.

**Fig. 3:**
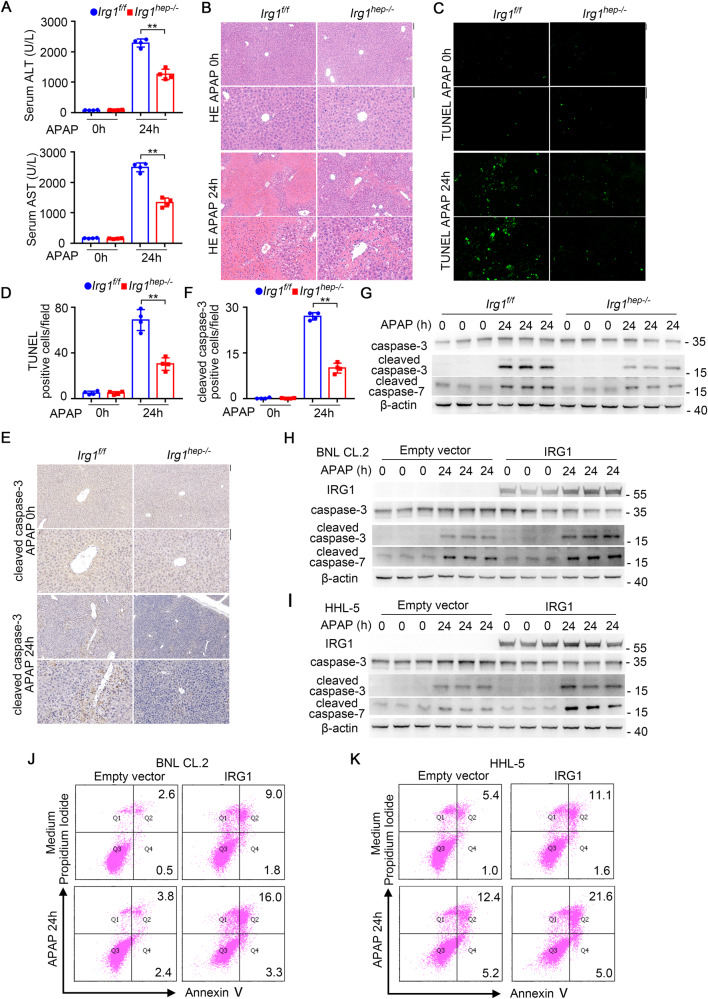
IRG1 promotes hepatotoxicant APAP-induced hepatocyte apoptosis and liver injury. **A**–**G** Eight-week-old male *Irg1*^*f/f*^ and *Irg1*^*hep-/-*^ mice were injected with APAP for the indicated time periods. APAP-induced liver damage was measured by serum ALT and AST (**A**, unpaired *t*-test). Liver pathology was analyzed by HE staining (**B**). Hepatic apoptosis was analyzed by TUNEL (**C**, **D**) and cleaved caspase-3 (**E**, **F**) staining (unpaired *t*-test), and western blot (**G**). Scale bars: 100 μm. **H**–**K** BNL CL.2 and HHL-5 hepatocyte cell lines were transfected with control vector or IRG1 overexpression plasmids, and treated with APAP for the indicated time periods. Hepatocyte apoptosis was analyzed by cleaved caspase-3/7 blotting (**H**, **I**) and flow cytometry (**J**, **K**). Data are shown as mean ± SD (*n* = 4) or photographs from one representative of three independent experiments. **P < 0.01.

The IRG1-promoted hepatocyte apoptosis was further confirmed in APAP-induced liver injury. The examination of serum ALT and AST, and analysis of hepatic pathology determined the suppressed liver damage in *Irg1*^*hep-/-*^ livers upon APAP injection (Fig. 3A, B). The APAP-induced apoptosis of hepatocytes was also found to be inhibited by hepatic IRG1 deficiency, shown by the reduced TUNEL staining and caspase-3/7 cleavage (Fig. 3C–G). Meanwhile, the infiltration of inflammatory cells was less influenced (Supplementary Fig. 3A–D). Furthermore, we overexpressed IRG1 in hepatocyte cell lines in vitro, and confirmed that IRG1 promoted APAP-induced apoptosis (Fig. 3H–K and Supplementary Fig. 3E–H). Together with the literatures determining hepatic apoptosis initiates hepatocarcinogenesis, we conclude that the induced IRG1 results in the apoptosis of hepatocytes and the promotion of HCC carcinogenesis.

### IRG1 promotes hepatocyte apoptosis independent of its product itaconate

**Fig. 4:**
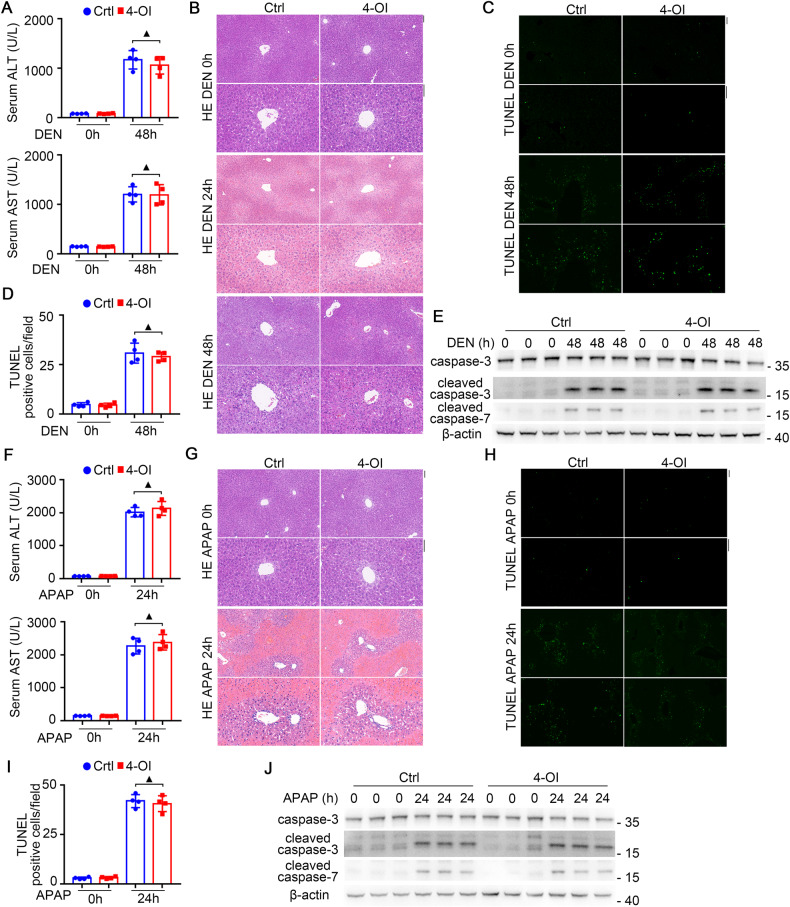
IRG1 promotes hepatocyte apoptosis independent of its product itaconate. Eight-week-old male mice were subjected to control or 4-OI intraperitoneal injection 2 h before DEN (**A**–**E**) or APAP (**F**–**J**) exposure. For DEN exposure, serum ALT and AST levels were analyzed (**A**, unpaired *t*-test), liver pathology was examined by HE staining (**B**), and hepatic apoptosis was analyzed by TUNEL (**C**, **D**) and cleaved caspase-3/7 (**E**) bloting as indicated. For APAP exposure, serum ALT and AST levels were analyzed (**F**, unpaired *t*-test), liver pathology was analyzed by HE staining (**G**), and hepatic apoptosis was analyzed by TUNEL (**H**, **I**) and cleaved caspase-3/7 (**J**) bloting as indicated. Scale bars: 100 μm. Data are shown as mean ± SD (*n* = 4) or photographs from one representative of three independent experiments. *P > 0.05.

As IRG1 is cis-aconitate decarboxylase to catalyze its substrate cis-aconitate and generate itaconate, and previous researches determined that IRG1 functions depended primarily on the product itaconate, we next examined whether IRG1-promoted apoptosis was dependent on its metabolite itaconate. The cell-permeable itaconate derivative 4-octyl itaconate (4-OI) was administrated, and both liver injury and hepatocyte apoptosis upon DEN injection were not influenced by 4-OI in vivo (Fig. 4A–E and Supplementary Fig. 4A, B). Similar results were also obtained in the model of APAP-induced liver injury (Fig. 4F–J and Supplementary Fig. 4C, D). Moreover, in 4-OI-treated hepatocyte cell lines, caspase-3/7 cleavage and apoptosis induction upon APAP administration were not influenced in vitro (Supplementary Fig. 4E–H). Thus, unlike previous knowledge that the function of IRG1 depends on itaconate, the induced IRG1-promoted hepatocyte apoptosis is independent on its product itaconate.

### IRG1 promotes the activation of mitochondrial intrinsic apoptosis

**Fig. 5:**
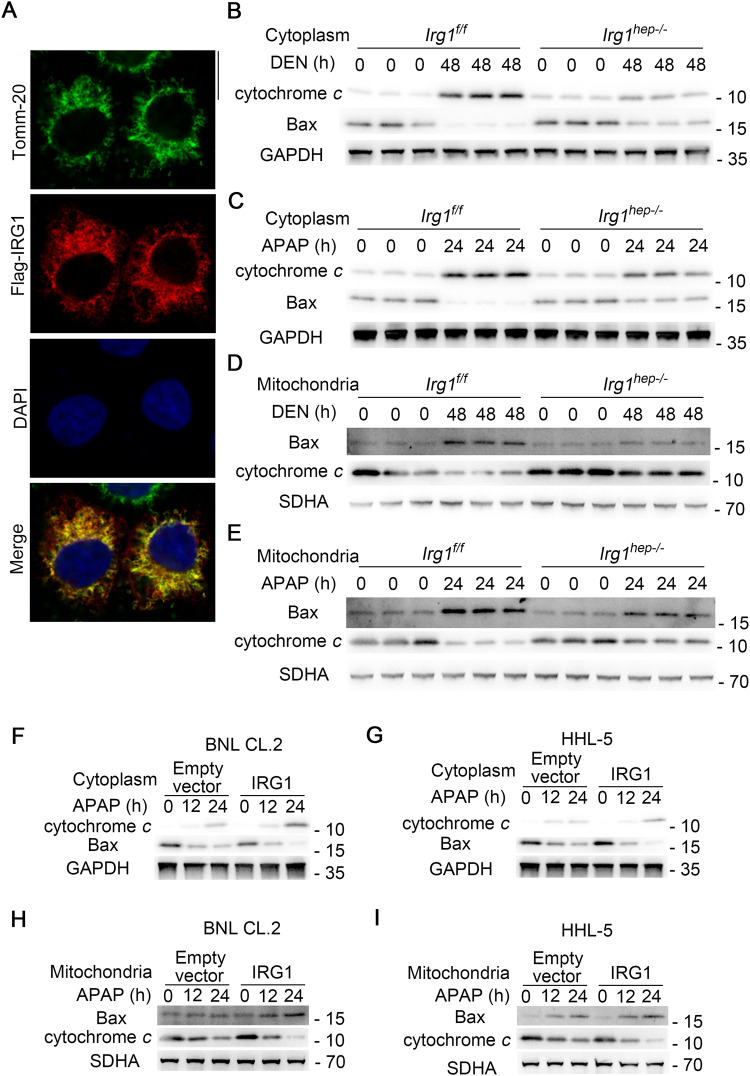
IRG1 promotes mitochondrial intrinsic apoptosis of hepatocytes. **A** Confocal microscopy of Tomm-20 (green), Flag-tagged IRG1 (red) and DAPI (blue). Scale bars: 10 μm. **B**, **C** Bax and the release of cytochrome *c* was examined in cytoplasmic proteins upon DEN (**B**) or APAP (**C**) stimulation as indicated. **D**, **E** The mitochondrial Bax and cytochrome *c* was examined in mitochondrial proteins upon DEN (**D**) or APAP (**E**) stimulation as indicated. **F**–**I** BNL CL.2 and HHL-5 hepatocyte cell lines were transfected with control vector or IRG1 overexpression plasmids, and treated with APAP for the indicated time periods. Bax and the release of cytochrome *c* was examined in cytoplasmic proteins upon APAP stimulation (**F**, **G**), and the mitochondrial Bax and cytochrome *c* was examined in mitochondrial proteins upon APAP stimulation as indicated (**H**, **I**). Data are shown as photographs from one representative of three independent experiments.

The underlying mechanism responsible for the induced IRG1-promoted hepatocyte apoptosis was then investigated. Since cleaved caspase-8 was the marker of death receptor-mediated extrinsic apoptosis [27], the DEN or APAP-induced cleavage of caspase-8 was not detected in IRG1 knockout mice or IRG1 overexpressing cells (Supplementary Fig. 5A–D), thus excluding the possibility that IRG1 promotes extrinsic apoptosis. Thereafter, we determined that IRG1 was colocalized with the mitochondrial outer membrane marker TOMM-20 in hepatocytes (Fig. 5A), suggesting that IRG1 may promote the activation of mitochondrial intrinsic apoptosis. The cytoplasmic cytochrome *c* was examined, and hepatic IRG1 deficiency markedly suppressed DEN or APAP-induced release of cytochrome *c* (Fig. 5B, C). Since the release of cytochrome *c* is mediated by the translocation of Bax from cytoplasm to permeabilize the outer membrane of mitochondrion, the DEN or APAP-induced mitochondrial translocation of Bax was significantly reduced in *Irg1*^*hep-/-*^ livers (Fig. 5D, E). Moreover, upon APAP administration, IRG1 overexpression also promoted the cytoplasmic release of cytochrome *c* and the mitochondrial translocation of Bax (Fig. 5F–I). Collectively, these data determine that the induced IRG1 promotes the mitochondrial translocation of Bax to activate the cytochrome *c* release-medicated intrinsic apoptosis.

### IRG1 promotes mitochondrial intrinsic apoptosis by binding and trapping anti-apoptotic MCL-1

**Fig. 6:**
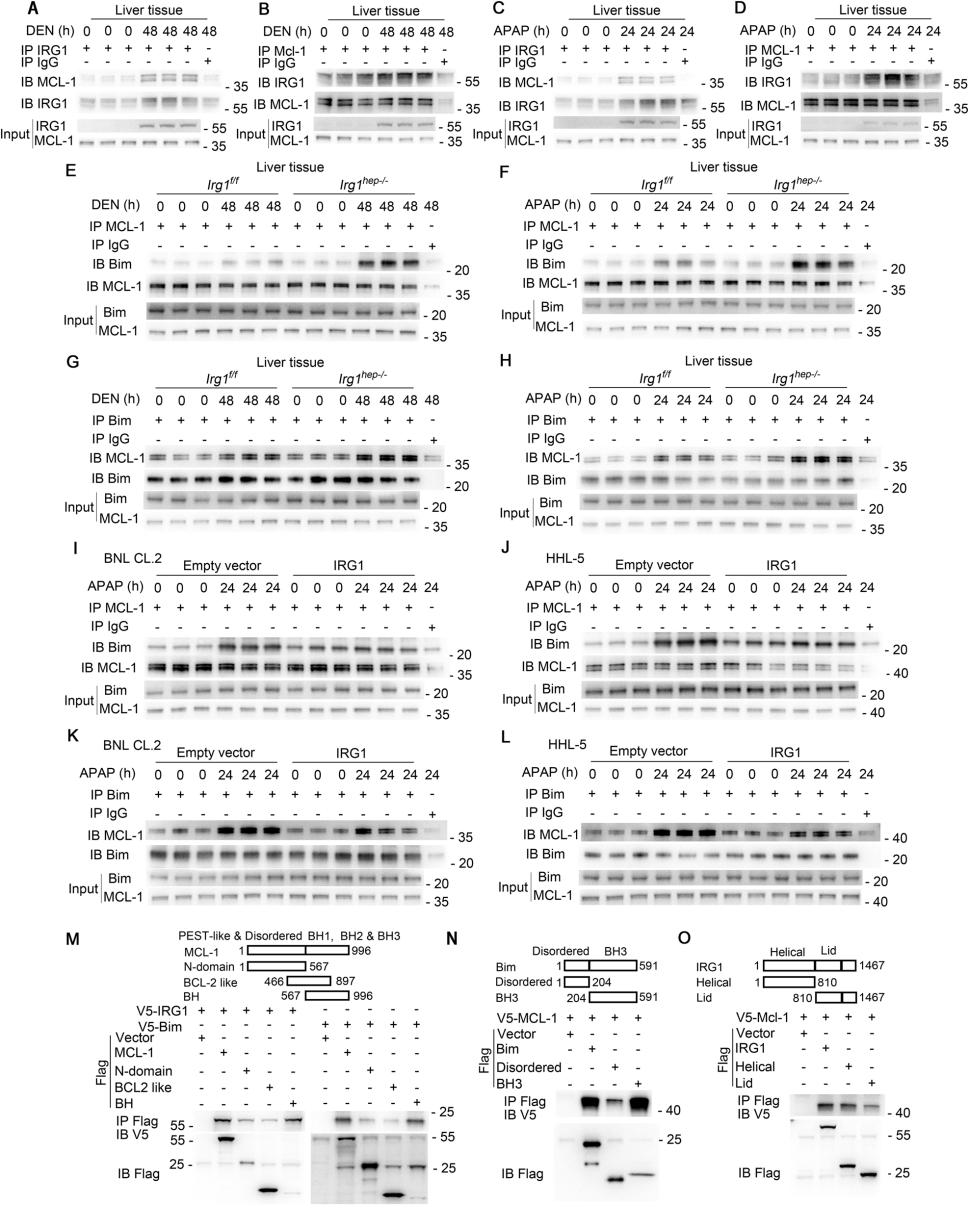
IRG1 promotes hepatocyte apoptosis by binding and trapping MCL-1. **A**–**D** Eight-week-old male mice were injected with DEN (**A**, **B**) or APAP (**C**, **D**), and the association between IRG1 and MCL-1 was examined by co-IP. **E**–**H** Eight-week-old male *Irg1*^*f/f*^ and *Irg1*^*hep-/-*^ mice were injected with DEN (**E**, **G**) or APAP (**F**, **H**) for the indicated time periods, and the association between MCL-1 and Bim was examined by co-IP. **I**–**L** BNL CL.2 (**I**, **K**) and HHL-5 (**J**, **L**) hepatocyte cell lines were transfected with control vector or IRG1 overexpression plasmid, and the association between MCL-1 and Bim was examined by co-IP. **M**–**O** Tagged IRG1, MCL-1, Bim and their truncates were constructed and co-transfected into HHL-5 cells, the cell lysates were precipitated with Flag antibody and immunoblotted with V5 antibody as indicated. Data are shown as photographs from one representative of three independent experiments.

**Fig. 7:**
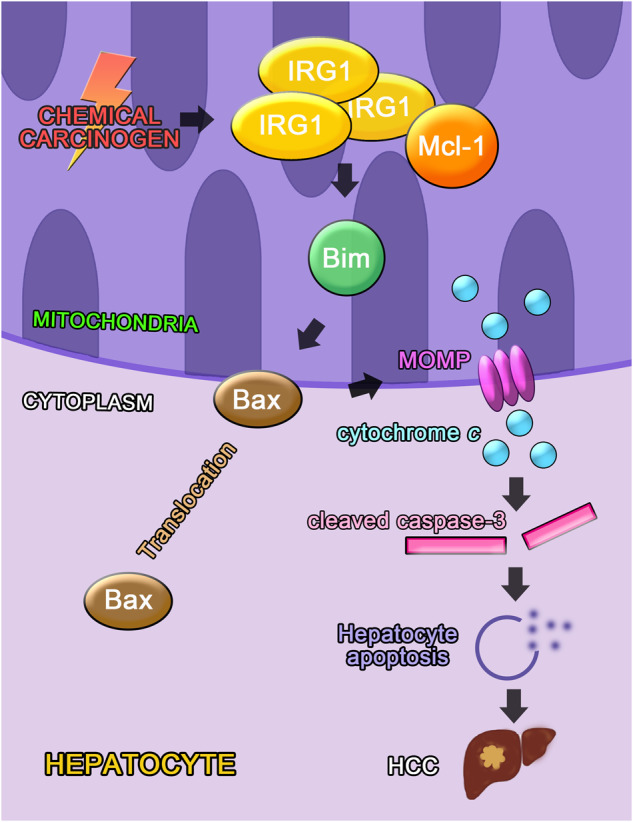
Working model for the mitochondrial IRG1 induced by chemical carcinogen for promoting hepatocyte apoptosis and the subsequent hepatocarcinogenesis. The DEN-induced mitochondrial IRG1 traps anti-apoptotic MCL-1 to inhibit the interaction between MCL-1 and pro-apoptotic Bim, and then promotes the activation of Bim and the mitochondrial translocation of downstream Bax, thus releasing cytochrome *c* to initiate intrinsic mitochondrial apoptosis and the subsequent hepatocarcinogenesis.

We further investigated the mechanism for IRG1-promoted mitochondrial translocation of Bax and activation of intrinsic apoptosis. As IRG1 promotes apoptosis independent on its metabolite itaconate, we analyzed whether IRG1 could directly interact with mitochondrial apoptotic proteins. Using immunoprecipitation coupled with mass spectrometry screening, IRG1 was found to be co-precipitated with anti-apoptotic MCL-1 (Supplementary Fig. 6A, B), which is a well-established pro-survival member of Bcl-2 family and determined to prevent apoptosis by sequestering the BH3 domain of pro-apoptotic proteins via BH3 groove. Moreover, knockout of MCL-1 in hepatocytes led to spontaneous HCC via exacerbating apoptosis [15]. The binding between IRG1 and MCL-1 was then confirmed, and their interaction was enhanced by DEN or APAP administration (Fig. 6A–D). MCL-1 is known to associate with pro-apoptotic Bim and inhibit the Bim-mediated mitochondrial translocation of Bax, thus suppressing apoptosis [13, 28]. And the mitochondrial translocation of Bax and the subsequent cytochrome *c* release were both confirmed here to be enhanced by MCL-1 knockdown while suppressed by Bim knockdown in hepatocytes (Supplementary Fig. 6C–H). Thereafter, we found that DEN or APAP-induced interaction between MCL-1 and Bim was significantly enhanced in *Irg1*^*hep-/-*^ livers (Fig. 6E–H). As the interaction between the shorter splice variants of Bim and MCL-1 was not significantly influenced by *Irg1*^*hep-/-*^ (data not shown), we only showed the longest Bim and MCL-1 association here. The overexpression of IRG1 in hepatocyte cell lines also inhibited the binding of MCL-1 and Bim (Fig. 6I–L). Furthermore, both the helical domain of IRG1 and the BH3 domain of Bim were found to interact with the BH region of MCL-1 (Fig. 6M–O and Supplementary Fig. 6I, J), suggesting the potential structural basis for the inhibited MCL-1-Bim interaction mediated by increased IRG1-MCL-1 association. Together, we conclude that the induced hepatic mitochondrial IRG1 traps anti-apoptotic MCL-1 to release pro-apoptotic Bim, thus enhancing the mitochondrial translocation of Bax to initiate cytochrome *c* release and intrinsic apoptosis, and the enhanced hepatocyte apoptosis medicated by the inducible IRG1 may ultimately lead to the promoted hepatocarcinogenesis (Fig. 7).

## Discussion

Cell death is fundamentally a driving force of almost every hepatic disease. For apoptosis of hepatocytes, the generated microenvironment is determined to induce the subsequent hepatocarcinogenesis [11]. Whereby, identifying the mechanistic insights of the intracellular regulatory mechanisms responsible for hepatocyte apoptosis and the subsequent carcinogenesis is essential to clarify the pathophysiology of HCC and develop new therapeutic interventions [9, 10, 29] In this study, we found that the chemical carcinogen-inducible IRG1 in hepatocytes promoted hepatic apoptosis and liver damage, which then facilitated hepatocarcinogenesis. Although the underlying mechanism for the induction of IRG1 in hepatocytes was still not determined here, the pro-apoptotic function of IRG1 was demonstrated to be mediated by the trapping of anti-apoptotic MCL-1 and the following initiation of intrinsic mitochondrial apoptosis, and the data show that the induced Bim-MCL-1 association during apoptosis, which may represent a feedback mechanism to inhibit apoptosis, is suppressed by the enhanced IRG1-MCL-1 interaction. Unlike the detrimental role of IRG1 in hepatocyte apoptosis, previous reports about IRG1 in liver macrophages and hepatocytes both suggested that IRG1 could protect liver against oxidative stress by activating NRF2 antioxidant response during hepatic lipid overload or ischemia-reperfusion injury [23, 24]. This discrepancy may be due to the different roles of IRG1 in the different intracellular signaling and biological processes. Furthermore, the previous reports of IRG1 suggested that IRG1 functions were dependent on its metabolite product itaconate [20, 30, 31], which is also different with its independence on itaconate in promoting apoptosis. Thus, other than the product itaconate, the function of IRG1 in the direct interaction with other proteins and pathways may raise interesting future works in this field.

For the potential mechanisms responsible for the pro-apoptotic role of IRG1 determined here and its protective role against ROS in previous reports, we speculate that the interaction between MCL-1 and IRG1 may compete with the enzymatic activity for converting cis-aconitate to itaconate, which may be also in accordance with the independence on itaconate to promote apoptosis and the dependence on itaconate to reduce ROS. Structurally, the region within IRG1 for its interaction with MCL-1 was identified to be the alike α-helical structure in its N-terminal helical domain, which was also responsible for the catalytic activity to produce itaconate, and the His 103 and Lys 207 located in this region was reported to be the key residues [32]. Thus, it is also possible that the IRG1 with itaconate-producing activity protects against oxidative stress at first, and the induced IRG1 with MCL-1 trapping activity promotes apoptosis at last. This presumption still needs further investigation in the future.

Considering the researches of IRG1 in carcinogenesis and progression, its roles in the progression of glioma have been suggested, which is the increased IRG1 in cancer cells promotes cell growth by enhancing cell cycle progression [33]. In addition, the augmented IRG1 and itaconate in peritoneal tissue-resident macrophage has also been associated with the promotion of peritoneal tumor cell growth by the boosted oxidative phosphorylation-driven ROS production in macrophages and ROS-mediated activation of MAPK in tumor cells, which leads to the enhanced peritoneal tumor progression [34]. Here, in normal cells such as hepatocytes, the induced IRG1 expression was determined to promote cell apoptosis, which is opposite to the pro-survival roles of IRG1 in malignant cells. The underlying mechanism is attracting, which may be also due to the possible preference of IRG1 in the production of itaconate to promote survival or in the interaction with MCL-1 to promote apoptosis. These presumptions are all based on the double-faced role of IRG1 in survival and apoptosis, and we will focus on this issue in our future study.

In the simplest model compatible with our findings, we propose that IRG1 expression in hepatocytes is markedly increased during liver damage, and the induced IRG1 promotes the apoptosis of hepatocytes and the subsequent hepatocarcinogenesis. Mechanistically, the induced mitochondrial IRG1 traps anti-apoptotic MCL-1 to inhibit the interaction between MCL-1 and pro-apoptotic Bim, and then promotes the activation of Bim and the mitochondrial translocation of downstream Bax, thus releasing cytochrome *c* to initiate intrinsic mitochondrial apoptosis (Fig. 7). Together, we find that the induced IRG1 in the mitochondrion during liver injury is an essential mechanism to promote the apoptosis of hepatocytes and the subsequent hepatocarcinogenesis.

## Materials and methods

### Animals

C57BL/6 mice were obtained from Joint Ventures Sipper BK Experimental Animal (Shanghai, China). *Irg1*^*f/f*^ mouse was constructed by Shanghai Biomodel Organism Science & Technology Development Cooperation (Shanghai, China). Alb-Cre transgenic mouse (003574) were obtained from The Jackson Laboratory. of Genomic DNA extraction and genotyping were described previously [35]. All animal experiments were conducted in accordance with National Institute of Health Guide for the Care and Use of Laboratory Animals, with the approval of the Scientific Investigation Board of Second Military Medical University, Shanghai, China.

### Reagents

Antibodies specific to human IRG1 (77510), mouse IRG1 (17805, 19857), caspase-3 (9662), cleaved caspase-3 (9661), caspase-8 (4790), human cleaved caspase-8 (9496), mouse cleaved caspase-8 (9429), cytochrome *c* (11940), SDHA (11998), Bax (2772), MCL-1 (94296), Bim (2933), Flag-tag (14793), V5-tag (13202) and horseradish peroxidase-coupled secondary antibodies (7074 and 7076) were from Cell Signaling Technology (Danvers, MA). Antibody specific to β-actin (A5441), DEN (N0258), CHX (239764), LPS (L3024), MEM NAA (M7145) and anti-Flag M2 magnetic beads (M8823) were from Sigma-Aldrich (St. Louis, MO). Recombinant human TNF-α (300-01A) and mouse TNF-α (315-01A) were from Pepro Tech (Rocky Hill, NJ). TUNEL assay kit (11684817910) was from Roche (Shanghai, China). Olive oil (CAS: 8001-25-0, A502795-0100) and APAP (CAS: 103-90-2, A506808) were from Sangon Biotech (Shanghai, China). Cell (C3601) and tissue (C3606) mitochondria isolation kits were from Beyotime (Shanghai, China). Percoll solution (17-0891-01) was from GE Healthcare Life Science (Little Chalfont, UK). Type IV collagenase (LS004140) was from Worthington Biochemical Corporation (Lakewood, NJ). Protease inhibitor cocktail (539134-1SML) and apoptosis assay kit (PF032) were from Calbiochem (Darmstadt, Germany). Fetal Bovine Serum (FBS, 10099141C), DMEM (11965092), and RPMI 1640 (11875093) were from Gibco (Shanghai, China). 4-OI (HY-112675) was from MedChemExpress (Monmouth Junction, NJ).

### Mouse models

For DEN-induced hepatocarcinogenesis model, 15-day-old male mice were randomly selected and given a single injection of DEN (25 mg/kg) intraperitoneally, and livers were harvested eight months later. For STAM HCC model, postnatal 2-day-old male mice were injected with STZ (200 ug per mouse), and these mice were fed with HFD (D12492, Research Diets, New Brunswick, NJ) at 1-month-old for 5 months. For acute liver injury model, 8-week-old male mice were randomly selected and treated with high-dose DEN (100 mg/kg) or APAP (400 mg/kg) intraperitoneally, and sacrificed at the indicated time points. For DEN-induced compensatory proliferation model, male mice at 15 days old were randomly selected and stimulated with low-dose DEN (25 mg/kg) via intraperitoneal injection, and livers were examined at the indicated time periods. 4-OI (25 mg/Kg, dissolved in olive oil) was injected intraperitoneally, and livers were examined as indicated.

### Cell lines and transfection

The human hepatocyte cell line HHL-5 and mouse hepatocyte cell line BNL CL.2 were both from the Type Culture Collection of the Chinese Academy of Sciences (Shanghai, China). HHL-5 was cultured in RPMI 1640 with 10% FBS, and BNL CL.2 was cultured in DMEM with 10% FBS and 1% non-essential amino acid. All cell lines were authenticated by STR profiling and tested for mycoplasma contamination by Genechem (Shanghai, China). Cells were seeded in culture dishes, and jetPRIME transfection reagent (114-15, Polyplus-transfection, France) was applied for plasmid transfection according to manufacturer’s protocols described previously [36].

### Primary hepatocyte isolation and treatment

Primary hepatocytes were isolated from 8-week-old mouse liver by the two-step liver perfusion and digestion methods described previously [37, 38]. After digestion, the liver capsule was peeled, minced and filtered with 70-micron membrane, then, primary hepatocytes were separated by density gradient centrifugation in 50% Percoll solution (P4937, Sigma-Aldrich) at 50 g for 5 min. The primary hepatocytes were resuspended in DMEM with 10% FBS and 1 × 10^7^ cells were seeded into six-well plates for attaching overnight.

### MS analysis

The IRG1-associated proteins were immunoprecipitated from cell lysates, and the precipitates were washed, boiled and loaded to SDS-PAGE. With Coomassie Blue staining, the specific bands in IRG1 lane were cut and analyzed in reverse-phase nanospray liquid chromatography-tandem mass spectrometry. The MS and spectra analysis were performed by PTM BIO (Hangzhou, China) as described previously [39].

### RNA extraction and real-time PCR

The total RNA were extracted by RNAiso Plus reagent (Takara, Dalian, China). Real-time quantitative RT-PCR (qRT-PCR) assay was performed by using LightCycler (Roche, Switzerland) and SYBR RT-PCR kit (RR430B, Takara, Dalian, China) as previously described [36]. The qPCR primers for detecting mRNA expression were mouse *IRG-1* (forward: 5’-ACT TCT CCA AGG AAG CCA AAG A-3’, reverse: 5’-ACT TTG TCA AGC TGA GCC CC-3’); internal control mouse *β-actin* (forward: 5’-AGT GTG ACG TTG ACA TCC GT-3’, reverse: 5’-GCA GCT CAG TAA CAG TCC GC-3’). The relative expression of each gene was normalized to the internal control using 2^-ΔΔCt^ cycle threshold method in each sample [40].

### Primer sequences for mice genotyping

*Irg1*^*f/f*^ forward: 5’-CTG AAA CTG TTA CCC TTA CAG-3’, reverse: 5’-AAG CTA GAT TGG CTT TAC AAT C-3’; Alb-cre forward: 5’-AAT GCT TCT GTC CGT TTG-3’, reverse: 5’-GGA TTA ACA TTC TCC CAC C-3’.

### Liver function assessment

Serum ALT and AST were assessed by using an automatic biochemical analyzer FDC-7000i (Shanghai, China) according to recommended instructions.

### Histological analysis

Tissues were fixed with paraformaldehyde and embedded in paraffin. Serial sections were sliced, and TUNEL, cleaved caspase-3, Ly6G, F4/80 and Ki67 staining were performed as previously described [36–38]. The quantification was measured using ImageJ software based on the positively stained cells and the intensity of the staining in representative sections.

### Immunoprecipitation and western blot

All samples were lysed in cell lysis buffer (9803, Cell Signaling Technology) with additional protease inhibitor cocktail (539134, Millipore) at a ratio of 1:200. Protein lysates concentration was measured by bicinchoninic acid assay kit (ab102536, Abcam) and equalized with lysis buffer. Equivalent amount of protein extracts was used for immunoprecipitation or loaded and subjected to SDS-PASGE, transferred onto nitrocellulose membranes, and then blotted as previously described [36, 37].

### Flow cytometry

Cells were collected and labeled with Annexin V-FITC/PI by using the apoptosis assay kit, and then subjected to flow cytometry analysis on BD Fortessa Cytometer and analyzed using FACSDiva software (Becton Dickinson).

### Statistical analysis

Data are shown as mean ± SD from one representative of three independent experiments. Statistical comparisons between groups were computed by unpaired Student’s *t*-test, *χ*^2^ test, or one-way ANOVA in SPSS 17.0 (Chicago, IL), and a two-tailed *P* < 0.05 indicates statistical significance.

### Reporting summary

Further information on research design is available in the Nature Research Reporting Summary linked to this article.

### Supplementary information

Supplementary Materials

Original Data

Reporting Summary

## Data Availability

All the unprocessed gels, images, and original source data for all figures are available at Mendeley Data Reserved https://data.mendeley.com/datasets/437w4z7w66/4.
