# Supplementary material for: Mitochondrial IRG1 traps MCL-1 to induce hepatocyte apoptosis and promote carcinogenesis
Source: Cell Death Dis. 2023 Sep 22;14(9):625. doi: 10.1038/s41419-023-06155-7 (PMC10517141; doi:10.1038/s41419-023-06155-7)
Supplement: Supplementary file 1 — Supplementary Materials [file 41419_2023_6155_MOESM1_ESM.docx]

**SUPPLEMENTARY MATERIALS**

**Mitochondrial IRG1 traps MCL-1 to induce hepatocyte apoptosis and promote carcinogenesis**

Liyuan Zhang, Yue Dong, Luxin Zhang, Minjun Wang, Ye Zhou, Kaiwei Jia, Suyuan Wang, Mu Wang, Yunhui Li, Shudan Luo, Shan Lu, Yiwen Fan, Dingji Zhang, Yingyun Yang, Nan Li, Yizhi Yu, Xuetao Cao and Jin Hou

Contents:

6 Supplementary Figures and Legends


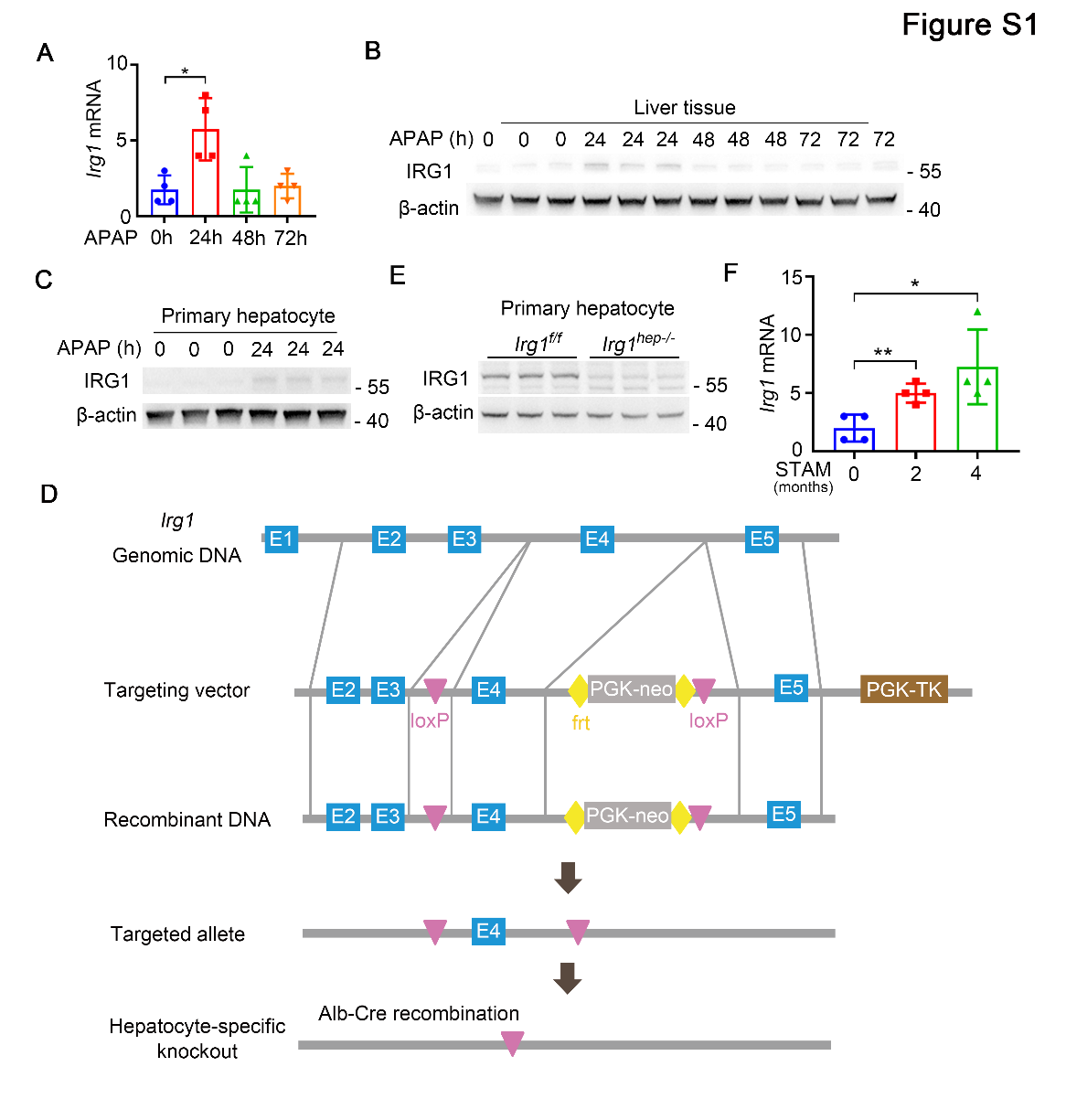


**Supplementary Fig. 1 The inducible expression of IRG1 in hepatocytes during liver injury and identification of IRG1 deficiency in *Irg1^hep-/-^* mice.**

**A, B** *Irg1* expression was examined by qRT-PCR (A) and Western blot (B) in the liver tissues from mice injected with APAP for the indicated time points (one-way ANOVA).

**C** APAP-induced IRG1 was examined by Western blot in the isolated hepatocytes from the mice treated as in B.

**D** Construction of hepatocyte-specific *Irg1* knockout mice.

**E** IRG1 expression in the isolated primary hepatocytes from *Irg1^f/f^* and *Irg1^hep-/-^* mice were confirmed using Western blot.

**F** *Irg1* mRNA expression in the STAM hepatocarcinogenesis mouse model was examined by qRT-PCR (one-way ANOVA).

Data are shown as mean ± s.d. (n=4) or photographs from one representative of three independent experiments. ***P* < 0.01, **P* < 0.05.

**
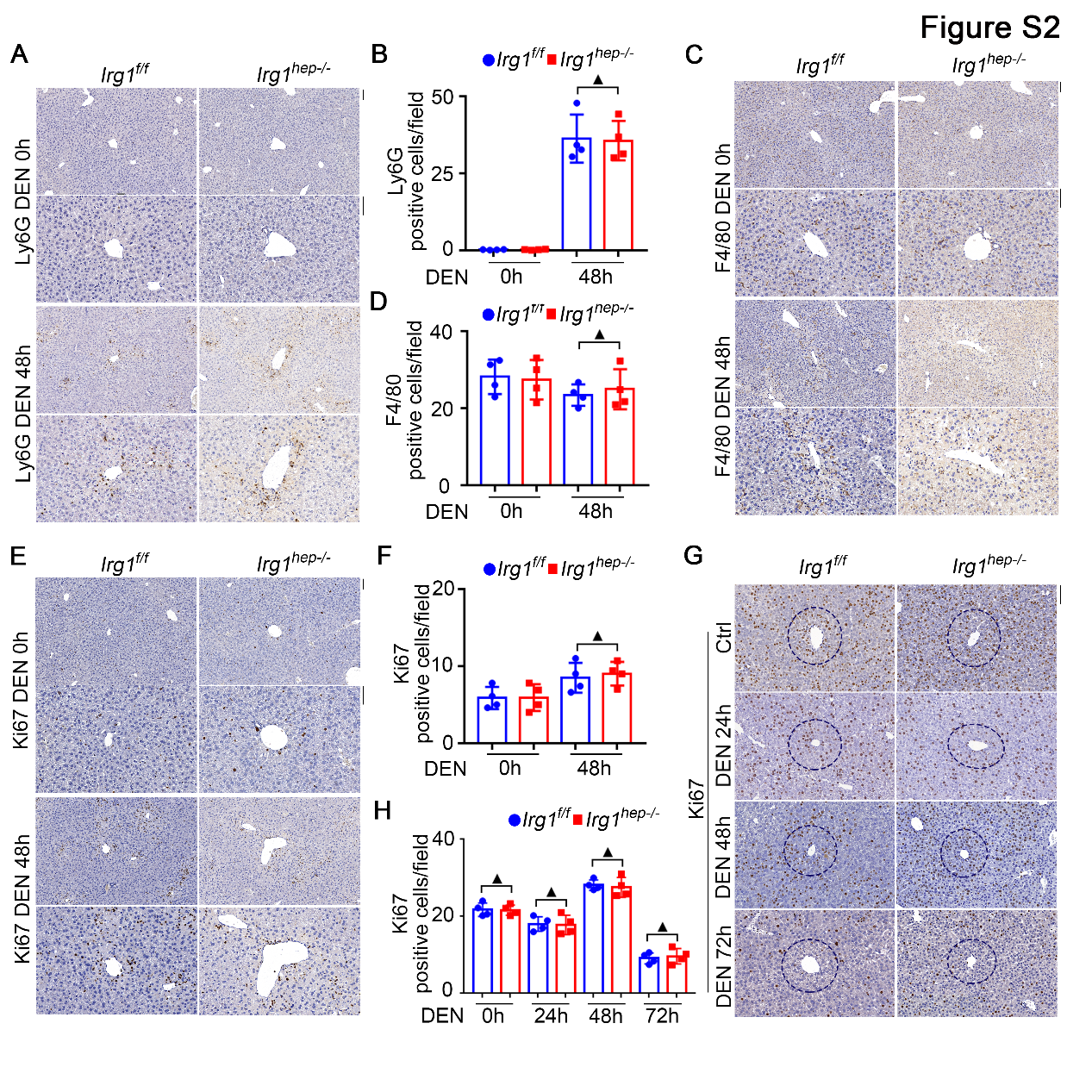
**

**Supplementary Fig. 2 The inducible IRG1 exacerbates DEN-induced liver injury.**

**A, B** Infiltrated leukocytes was analyzed by Ly6G staining (A) and quantified (B, unpaired *t*-test).

**C, D** Infiltrated macrophages was analyzed by F4/80 staining (C) and quantified (D, unpaired *t*-test).

**E-H** Compensatory proliferation of hepatocytes was analyzed by Ki67 staining and quantified in adult mice (E, F, unpaired *t*-test) and 15-day-old mice (G, H, unpaired *t*-test) respectively in the indicated time points post DEN injection. Scale bars: 100 μm.

Data are shown as mean ± s.d. (n=4) or photographs from one representative of three independent experiments. ^▲^*P* > 0.05.


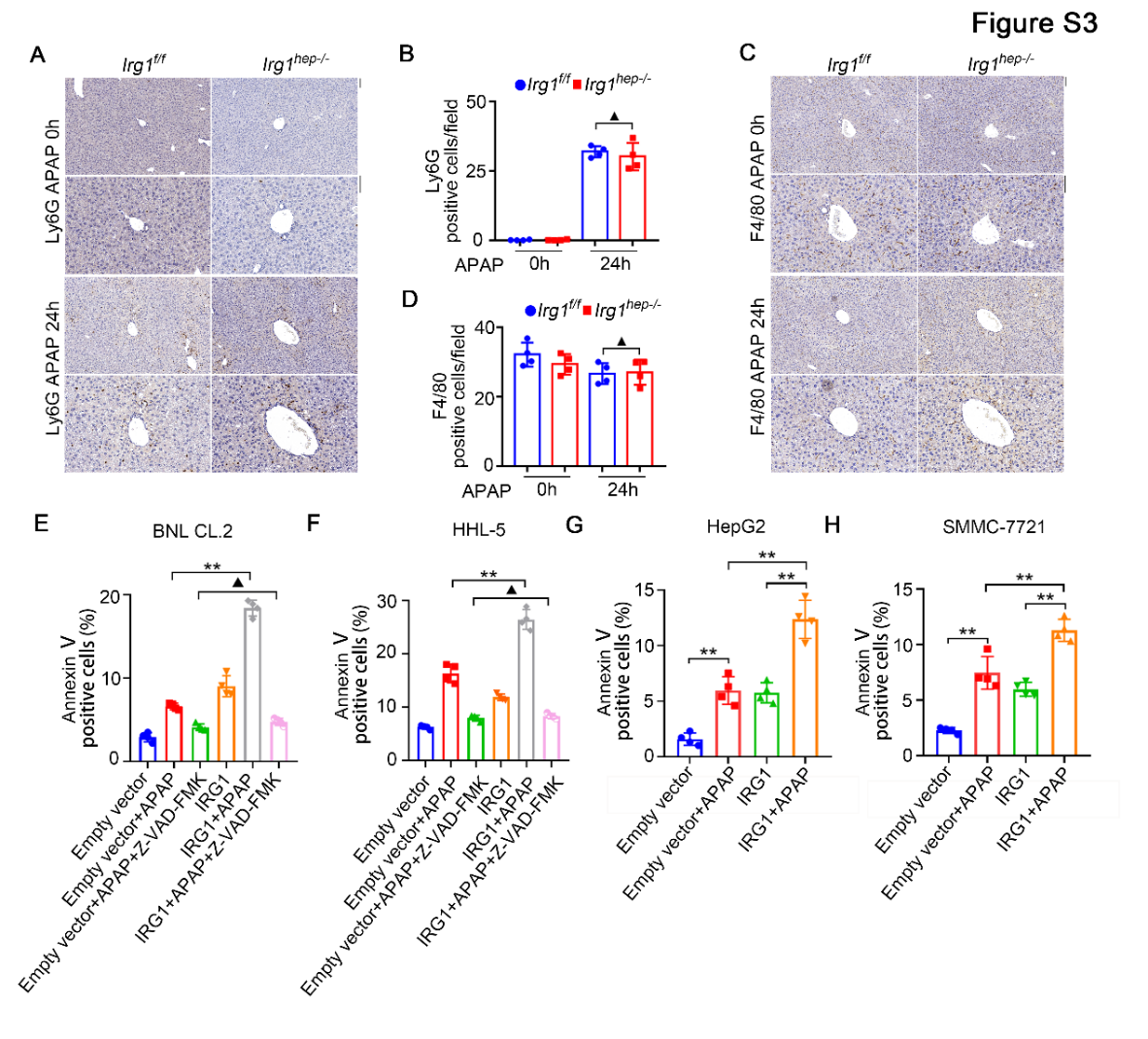


**Supplementary Fig. 3 IRG1 aggravates hepatotoxicant APAP-induced hepatocyte apoptosis and liver injury.**

**A, B** Infiltrated leukocytes was analyzed by Ly6G staining (A) and quantified (B, unpaired *t*-test).

**C, D** Infiltrated macrophages was analyzed by F4/80 staining (C) and quantified (D, unpaired *t*-test). Scale bars: 100 μm.

**E, F** BNL CL.2 and HHL-5 hepatocyte cell lines were transfected with control vector or IRG1 overexpression plasmid, and treated with APAP (and Z-VAD-FMK as indicated) for 24 hours. Hepatocyte apoptosis was analyzed by flow cytometry (unpaired *t*-test).

**G, H** HepG2 and SMMC-7721 cells were transfected with control vector or IRG1 overexpression plasmid, and then administrated with APAP for 24 hours. Cell apoptosis was analyzed by flow cytometry (unpaired t-test).

Data are shown as mean ± s.d. (n=4) or photographs from one representative of three independent experiments. ***P* < 0.01, ^▲^*P* > 0.05.


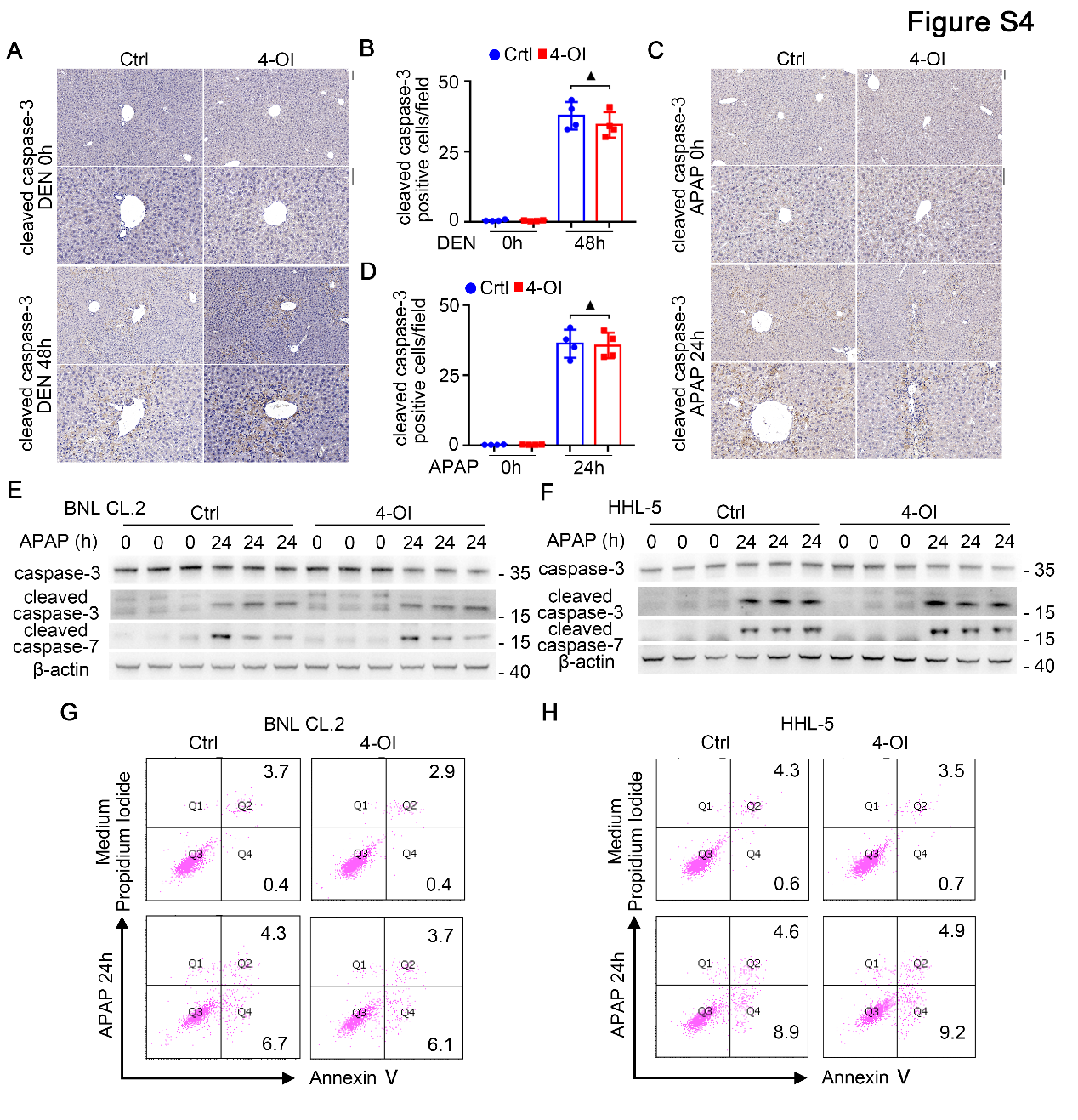


**Supplementary Fig. 4** **IRG1-produced itaconate does not influence DEN or APAP-induced hepatocyte apoptosis.**

**A-D** Eight-week-old male mice were subjected to control or 4-OI intraperitoneal injection two hours before DEN (A, B) or APAP (C, D) exposure, hepatic apoptosis was analyzed by cleaved caspase-3 staining and then quantified (unpaired *t*-test). Scale bars: 100 μm.

**E-H** BNL CL.2 and HHL-5 cell lines were treated with control or 4-OI two hours before APAP stimulation. Hepatic apoptosis was analyzed by cleaved caspase-3/7 blotting (E, F) and flow cytometry (G, H).

Data are shown as mean ± s.d. (n=4) or photographs from one representative of three independent experiments. ^▲^*P* > 0.05.

**
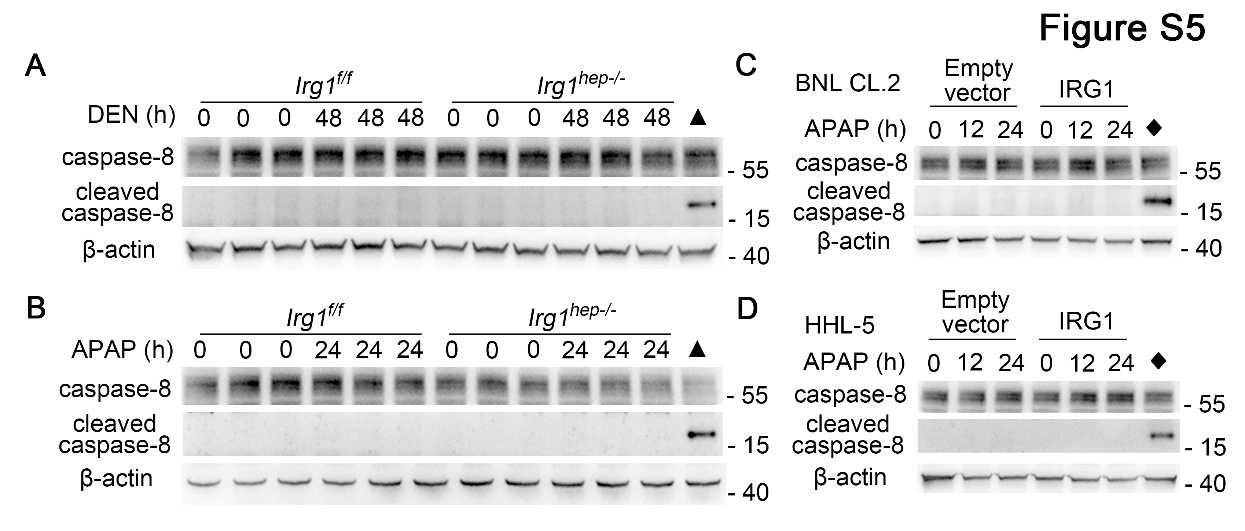
**

**Supplementary Fig. 5 IRG1 promotes hepatocyte apoptosis independent of the extrinsic apoptotic pathway.**

**A, B** Eight-week-old male *Irg1^f/f^* and *Irg1^hep-/-^* mice were injected with DEN (A) or APAP (B) for the indicated time periods, and cleaved caspase-8 was measured.

**C, D** BNL CL.2 (C) and HHL-5 (D) hepatocyte cell lines were transfected with control vector or IRG1 overexpression plasmid, and treated with APAP for the indicated time periods, and cleaved caspase-8 was measured by Western blot.

Data are shown as photographs from one representative of three independent experiments. ▲, LPS+D-gal; ◆, TNF-α+CHX; positive controls.

**
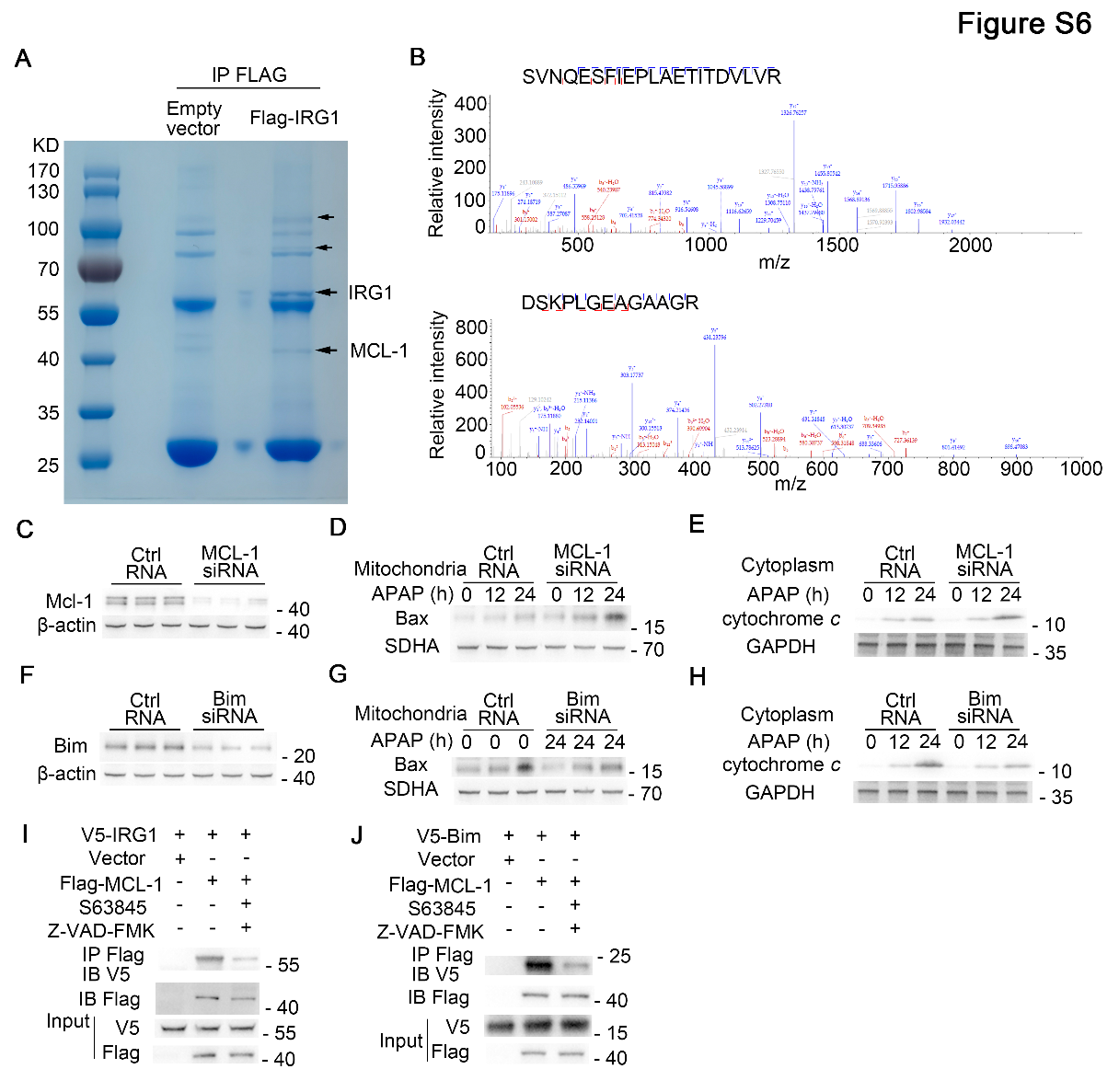
**

**Supplementary Fig. 6 The screen of IRG1-associating proteins using IP-MS.**

**A** Empty vector or Flag-tagged IRG1 plasmid were transfected into HHL-5 hepatocyte cell line, and PAGE gel resolution of immunoprecipitated Flag-tag and its binding proteins. The arrows indicate three different bands selected for further MS analysis, and MCL-1 band is detected as indicated.

**B** Tandem mass spectrometry spectrum of MCL-1.

**C-H** HHL-5 cells were transfected with control, MCL-1 siRNA (C) or Bim siRNA (F) as indicated. Bax in mitochondria (D, G) and cytochrome *c* release (E, H) upon APAP administration were examined by Western blot.

**I, J** Tagged IRG1, Bim and MCL-1 were co-transfected into HHL-5 cells, and treated with S63845 and Z-VAD-FMK as indicated, the cell lysates were precipitated with Flag antibody and immunoblotted with V5 antibody.

Data are shown as photographs from one representative of three independent experiments.
