# Supplementary material for: Mitochondrial IRG1 traps MCL-1 to induce hepatocyte apoptosis and promote carcinogenesis
Source: Cell Death Dis. 2023 Sep 22;14(9):625. doi: 10.1038/s41419-023-06155-7 (PMC10517141; doi:10.1038/s41419-023-06155-7)
Supplement: Supplementary file 2 — Original Data [file 41419_2023_6155_MOESM2_ESM.docx]

**Figure 1A**

| C57BL/6 mice (n=4) | *β-actin* Ct value | *Irg1* Ct value | 2^-∆∆Ct^ |
| --- | --- | --- | --- |
| DEN 0h-1 | 14.587 | 29.643 | 0.00002 |
| DEN 0h-2 | 15.916 | 30.514 | 0.00004 |
| DEN 0h-3 | 16.081 | 30.443 | 0.00004 |
| DEN 0h-4 | 15.945 | 30.532 | 0.00004 |
|  |  |  |  |
| DEN 24h-1 | 13.934 | 30.340 | 0.00001 |
| DEN 24h-2 | 14.609 | 29.928 | 0.00002 |
| DEN 24h-3 | 14.669 | 29.786 | 0.00002 |
| DEN 24h-4 | 14.248 | 29.429 | 0.00002 |
|  |  |  |  |
| DEN 48h-1 | 14.617 | 27.608 | 0.00012 |
| DEN 48h-2 | 14.159 | 27.685 | 0.00008 |
| DEN 48h-3 | 15.255 | 28.474 | 0.00010 |
| DEN 48h-4 | 14.397 | 27.775 | 0.00009 |
|  |  |  |  |
| DEN 72h-1 | 12.765 | 26.741 | 0.00006 |
| DEN 72h-2 | 12.436 | 27.701 | 0.00002 |
| DEN 72h-3 | 12.375 | 27.863 | 0.00002 |
| DEN 72h-4 | 12.679 | 28.086 | 0.00002 |

*P* < 0.001 (**)

**Figure 1B**

IRG1





β-actin





**Figure 1C**

IRG1





β-actin





**Figure 1D**

*Irg1^f/f^*

*^
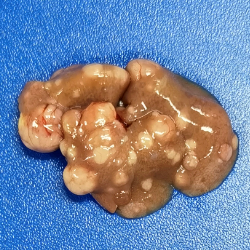

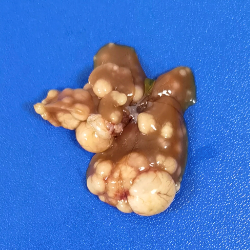
^*

*Irg1^hep-/-^*

*^
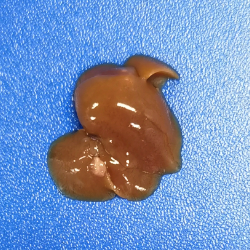

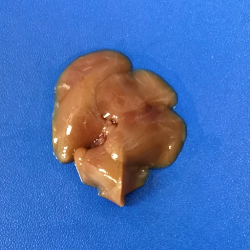
^*

**Figure 1E**

Tumor incidence (%)

| *Irg1^f/f^* (n=15) | *Irg1^hep-/-^* (n=15) |
| --- | --- |
| 80 (12/15) | 33.33 (5/15) |

*P* = 0.028 (*)

Tumor number per mouse

| *Irg1^f/f^* (n=15) | *Irg1^hep-/-^* (n=15) |
| --- | --- |
| 17 | 0 |
| 0 | 3 |
| 30 | 0 |
| 12 | 2 |
| 0 | 0 |
| 3 | 2 |
| 15 | 0 |
| 4 | 7 |
| 2 | 0 |
| 8 | 10 |
| 8 | 0 |
| 30 | 0 |
| 0 | 0 |
| 6 | 0 |
| 29 | 0 |

*P* = 0.006 (**)

Maximal tumor diameter per mouse (mm)

| *Irg1^f/f^* (n=15) | *Irg1^hep-/-^* (n=15) |
| --- | --- |
| 9.90 | 0 |
| 0 | 8.15 |
| 8.72 | 0 |
| 6.75 | 2.99 |
| 0 | 0 |
| 1.6 | 1.89 |
| 3.93 | 0 |
| 2.17 | 5.6 |
| 3.25 | 0 |
| 3.58 | 6.81 |
| 8.04 | 0 |
| 9.36 | 0 |
| 0 | 0 |
| 6.09 | 0 |
| 14.23 | 0 |

*P* = 0.014 (*)

**Figure 1F**

*Irg1^f/f^*


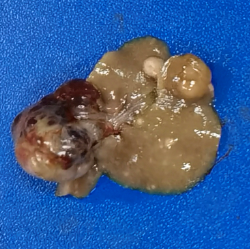

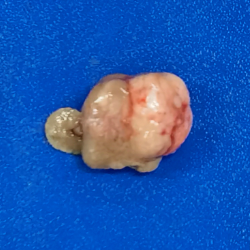


*Irg1^hep-/-^*


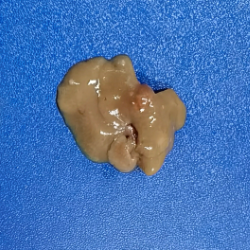

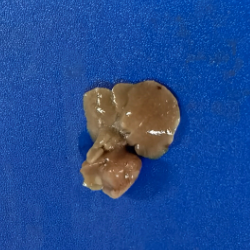


**Figure 1G**

Tumor incidence (%)

| *Irg1^f/f^* (n=15) | *Irg1^hep-/-^* (n=15) |
| --- | --- |
| 80 (12/15) | 40 (6/15) |

*P* = 0.021 (*)

Tumor number per mouse

| *Irg1^f/f^* (n=15) | *Irg1^hep-/-^* (n=15) |
| --- | --- |
| 8 | 0 |
| 3 | 0 |
| 3 | 3 |
| 0 | 0 |
| 0 | 5 |
| 10 | 0 |
| 16 | 3 |
| 8 | 8 |
| 12 | 0 |
| 17 | 0 |
| 4 | 5 |
| 0 | 0 |
| 6 | 0 |
| 7 | 1 |
| 9 | 0 |

*P* = 0.003 (**)

Maximal tumor diameter per mouse (mm)

| *Irg1^f/f^* (n=15) | *Irg1^hep-/-^* (n=15) |
| --- | --- |
| 20.15 | 0 |
| 17.27 | 0 |
| 7.99 | 11.38 |
| 0 | 0 |
| 0 | 0.28 |
| 18.82 | 0 |
| 7.66 | 1.14 |
| 21.01 | 7.29 |
| 13.94 | 0 |
| 9.07 | 0 |
| 16.95 | 4.23 |
| 0 | 0 |
| 14.44 | 0 |
| 5.85 | 2.91 |
| 19.78 | 0 |

*P* < 0.001 (**)

**Figure S1A**

| C57BL/6 mice (n=4) | *β-actin* Ct value | *Irg1* Ct value | 2^-∆∆Ct^ |
| --- | --- | --- | --- |
| APAP 0h-1 | 14.945 | 29.904 | 0.00003 |
| APAP 0h-2 | 14.592 | 30.683 | 0.00001 |
| APAP 0h-3 | 14.112 | 30.196 | 0.00001 |
| APAP 0h-4 | 14.245 | 29.92 | 0.00002 |
|  |  |  |  |
| APAP 24h-1 | 13.209 | 26.976 | 0.00007 |
| APAP 24h-2 | 13.052 | 27.580 | 0.00004 |
| APAP 24h-3 | 13.190 | 27.496 | 0.00004 |
| APAP 24h-4 | 13.204 | 26.670 | 0.00008 |
|  |  |  |  |
| APAP 48h-1 | 13.902 | 28.581 | 0.00004 |
| APAP 48h-2 | 14.003 | 30.205 | 0.00001 |
| APAP 48h-3 | 13.203 | 29.347 | 0.00001 |
| APAP 48h-4 | 13.028 | 29.720 | 0.00001 |
|  |  |  |  |
| APAP 72h-1 | 14.929 | 30.948 | 0.00002 |
| APAP 72h-2 | 13.846 | 28.728 | 0.00003 |
| APAP 72h-3 | 13.746 | 29.067 | 0.00002 |
| APAP 72h-4 | 14.832 | 31.369 | 0.00001 |

*P* = 0.02 (*)

**Figure S1B**

IRG1





β-actin





**Figure S1C**

IRG1





β-actin





**Figure S1D**


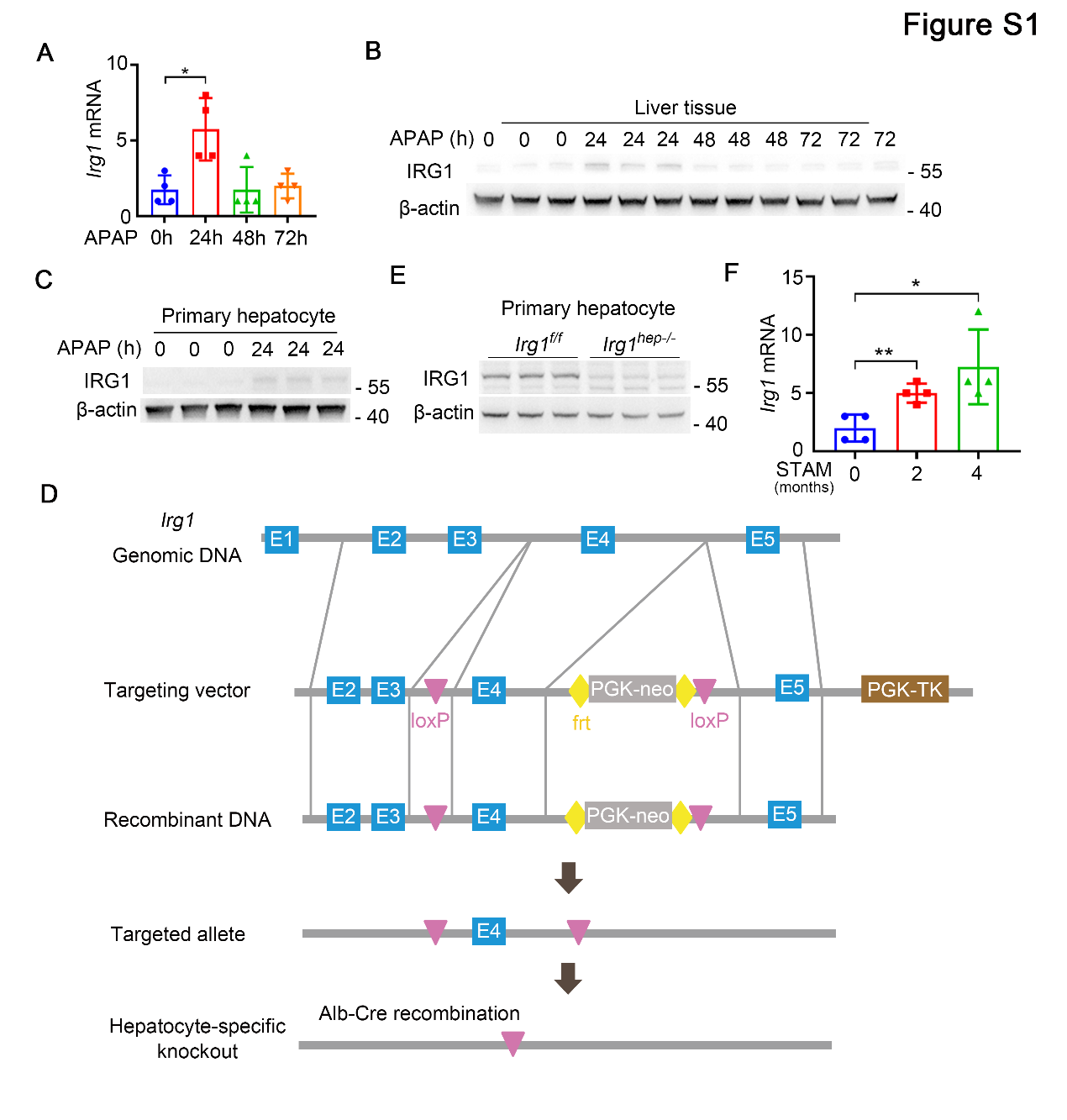


**Figure S1E**

IRG1





β-actin





**Figure S1F**

| C57BL/6 mice (n=4) | *β-actin* Ct value | *Irg1* Ct value | 2^-∆∆Ct^ |
| --- | --- | --- | --- |
| 0 month | 16.254 | 31.285 | 0.00003 |
| 0 month | 15.034 | 31.481 | 0.00001 |
| 0 month | 15.727 | 30.796 | 0.00003 |
| 0 month | 16.325 | 32.748 | 0.00001 |
|  |  |  |  |
| 2 months | 16.199 | 30.410 | 0.00005 |
| 2 months | 16.850 | 31.402 | 0.00004 |
| 2 months | 16.336 | 30.756 | 0.00005 |
| 2 months | 15.609 | 29.717 | 0.00006 |
|  |  |  |  |
| 4 months | 16.008 | 29.026 | 0.00012 |
| 4 months | 16.604 | 30.574 | 0.00006 |
| 4 months | 15.420 | 29.414 | 0.00006 |
| 4 months | 16.005 | 30.28 | 0.00005 |

**Figure 2A**

Serum ALT (U/L)

| Control | | DEN 48h | |
| --- | --- | --- | --- |
| *Irg1^f/f^* | *Irg1^hep-/-^* | *Irg1^f/f^* | *Irg1^hep-/-^* |
| 79.4 | 86.8 | 1108.9 | 508.9 |
| 83.3 | 78.5 | 1030.7 | 600.5 |
| 87.5 | 82.4 | 1208.0 | 798.2 |
| 85.9 | 74.0 | 1262.9 | 751.4 |

*P* = 0.006 (**)

Serum AST (U/L)

| Control | | DEN 48h | |
| --- | --- | --- | --- |
| *Irg1^f/f^* | *Irg1^hep-/-^* | *Irg1^f/f^* | *Irg1^hep-/-^* |
| 146.2 | 144.8 | 1290.9 | 765.8 |
| 169.6 | 179.2 | 1368.9 | 700.2 |
| 172.9 | 158.6 | 1195.4 | 502.0 |
| 152.6 | 168.4 | 1402.6 | 896.8 |

*P* = 0.001 (**)

**Figure 2B**

*Irg1^f/f^* DEN 0h-HE

**
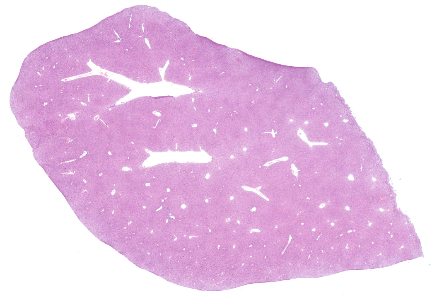

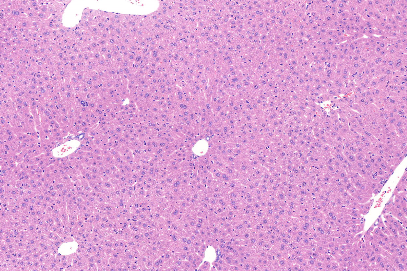

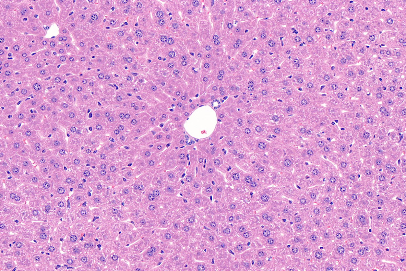
**

*Irg1^hep-/-^* DEN 0h-HE

**
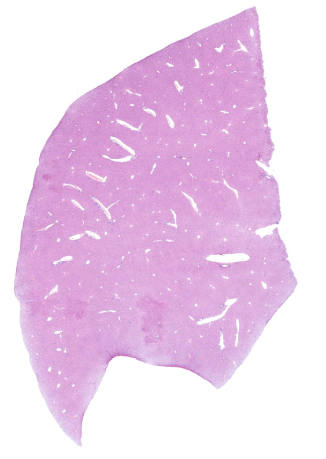

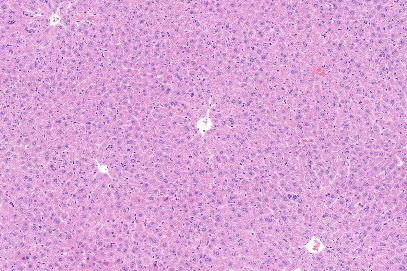

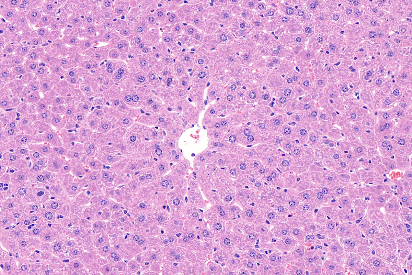
**

*Irg1^f/f^* DEN 24h-HE

*
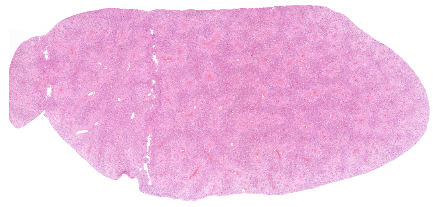

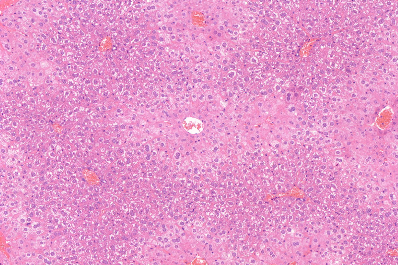

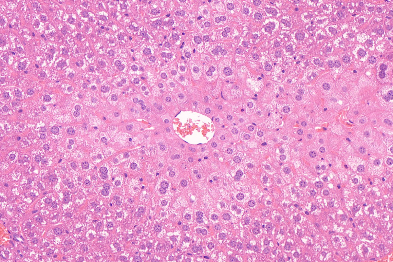
*

*Irg1^hep-/-^* DEN 24h-HE

*
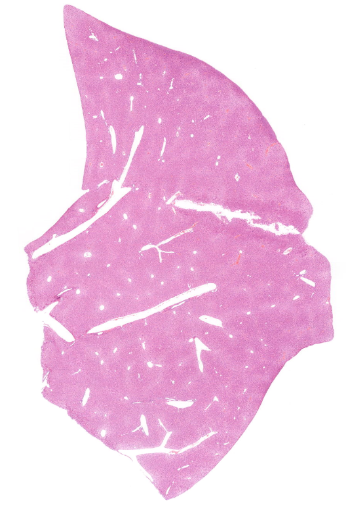

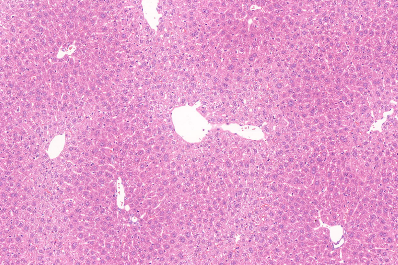

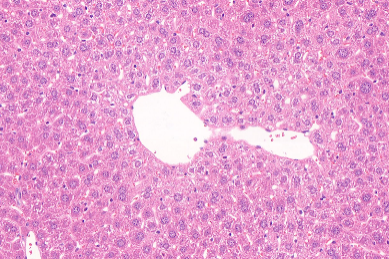
*

*Irg1^f/f^* DEN 48h-HE

**
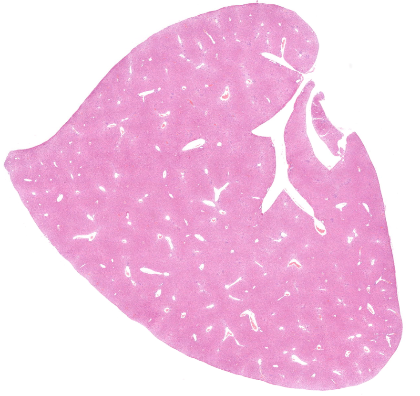

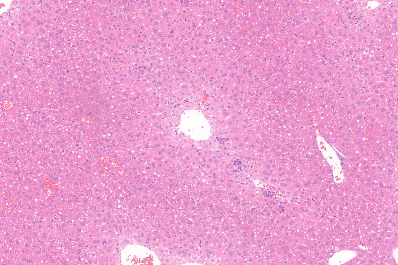

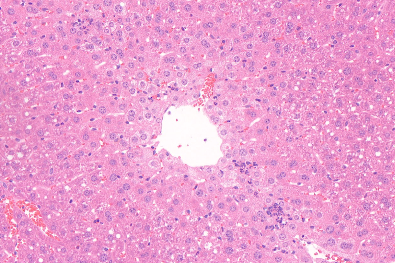
**

*Irg1^hep-/-^* DEN 48h-HE

**
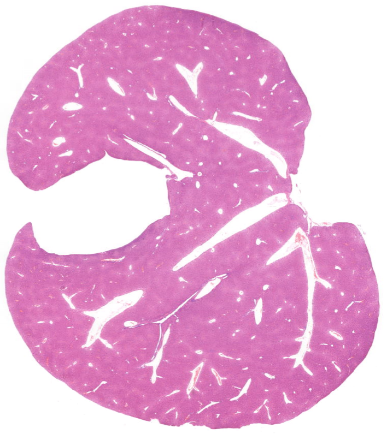

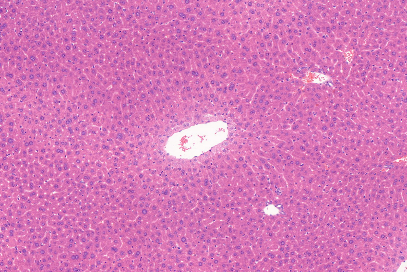

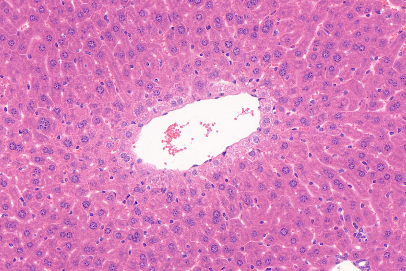
**

**Figure 2C**

*Irg1^f/f^* DEN 0h-TUNEL

**
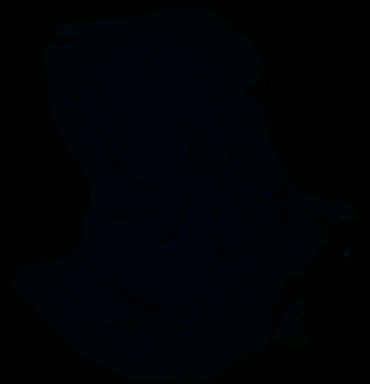

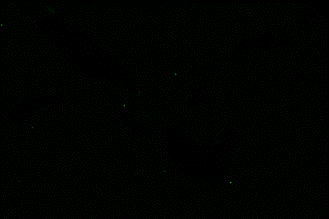

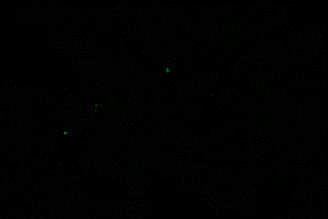
**

*Irg1^hep-/-^* DEN 0h-TUNEL

**
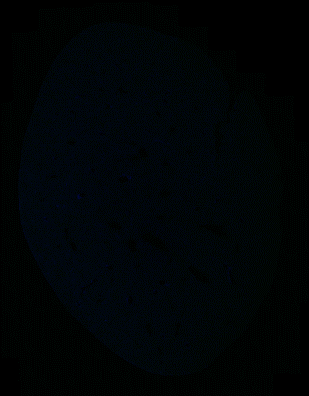

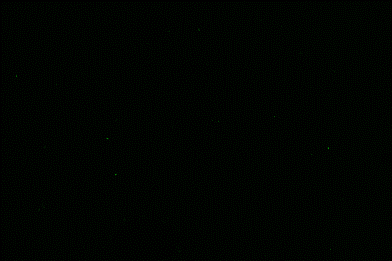

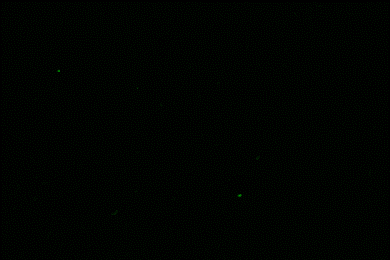
**

*Irg1^f/f^* DEN 48h-TUNEL

**
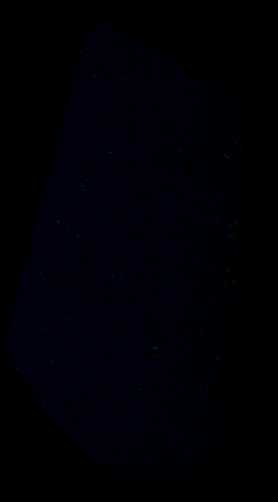

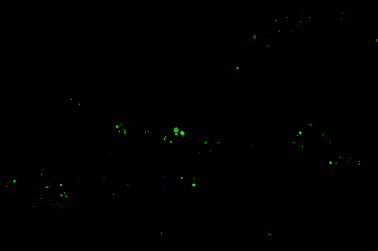

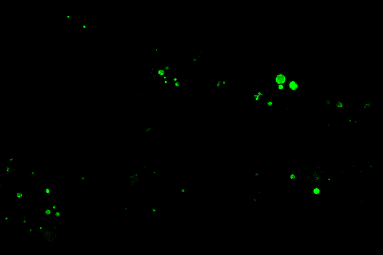
**

*Irg1^hep-/-^* DEN 48h-TUNEL

**
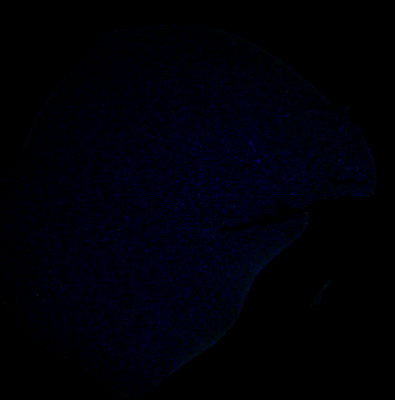

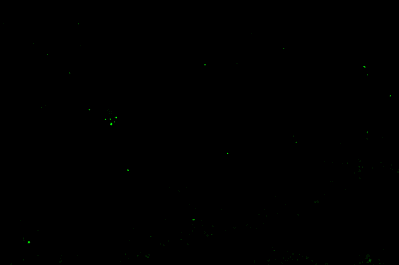

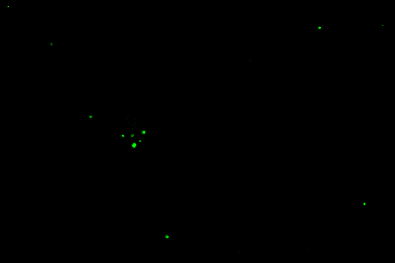
**

**Figure 2D**

| Control | | DEN 48h | |
| --- | --- | --- | --- |
| *Irg1^f/f^* | *Irg1^hep-/-^* | *Irg1^f/f^* | *Irg1^hep-/-^* |
| 2.70 | 1.80 | 56.27 | 30.00 |
| 3.00 | 2.40 | 60.96 | 22.83 |
| 2.20 | 2.80 | 49.34 | 34.90 |
| 2.40 | 2.40 | 66.29 | 38.88 |

*P* = 0.002 (**)

**Figure 2E**

*Irg1^f/f^* DEN 0h-cleaved caspase-3

**
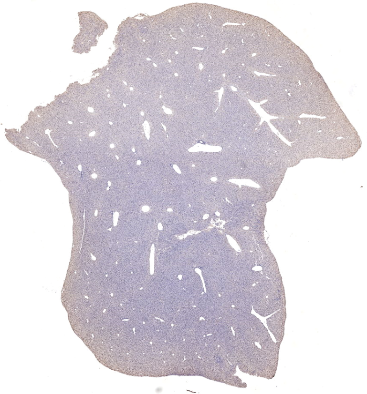

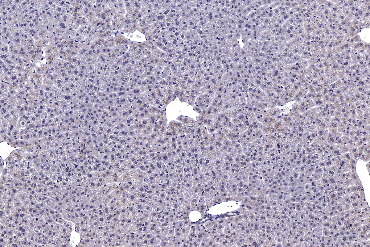

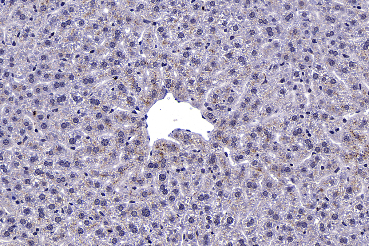
**

*Irg1^hep-/-^* DEN 0h-cleaved caspase-3

**
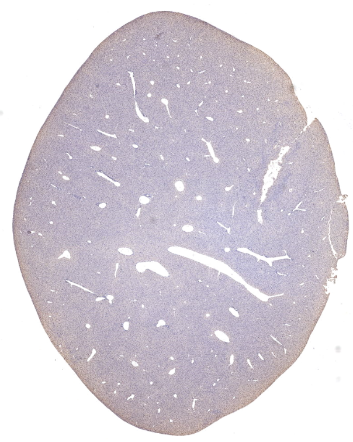

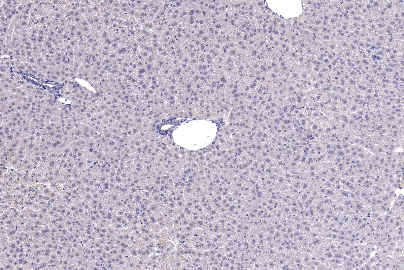

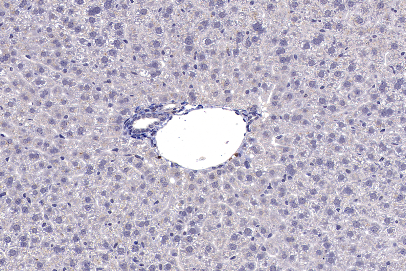
**

*Irg1^f/f^* DEN 48h-cleaved caspase-3

**
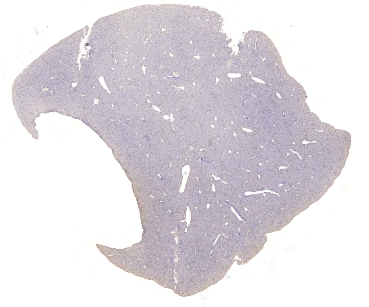

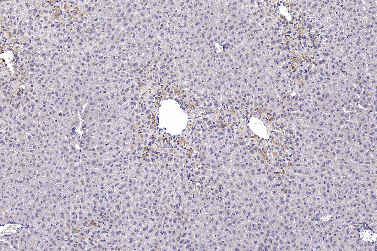

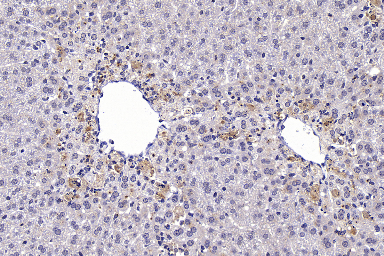
**

*Irg1^hep-/-^* DEN 48h-cleaved caspase-3

**
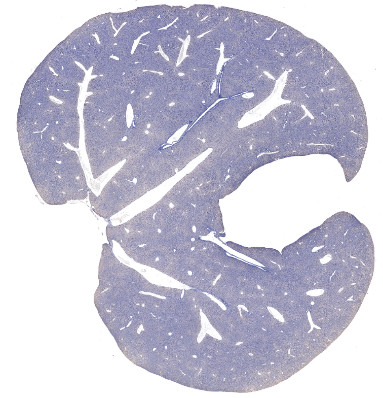

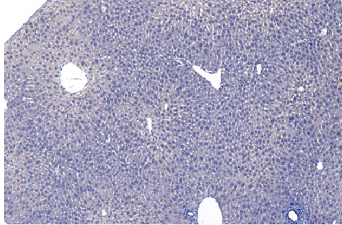

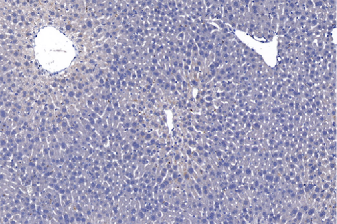
**

**Figure 2F**

Quantification

| Control | | DEN 48h | |
| --- | --- | --- | --- |
| *Irg1^f/f^* | *Irg1^hep-/-^* | *Irg1^f/f^* | *Irg1^hep-/-^* |
| 0 | 0.3 | 24.20 | 11.00 |
| 0 | 0 | 22.90 | 9.80 |
| 0.2 | 0.2 | 21.00 | 10.20 |
| 0.2 | 0 | 20.20 | 12.80 |

*P* < 0.001 (**)

**Figure 2G**

caspase-3





cleaved caspase-3





cleaved caspase-7





β-actin



 **Figure S2A**

*Irg1^f/f^* DEN 0h-Ly6G

**
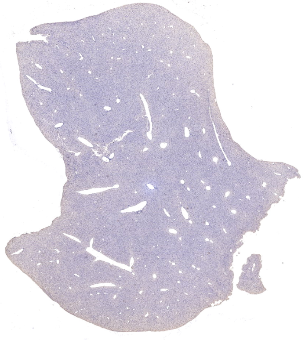

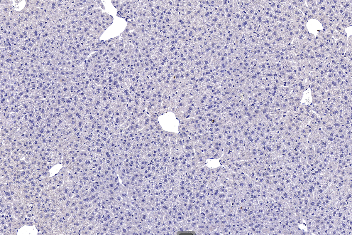

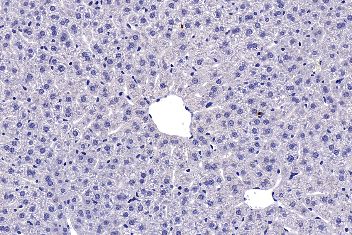
**

*Irg1^hep-/-^* DEN 0h-Ly6G

**
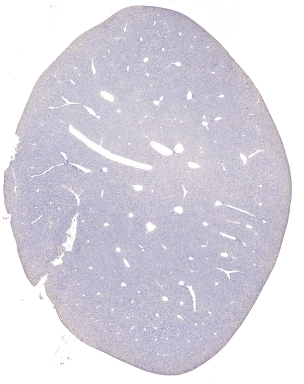

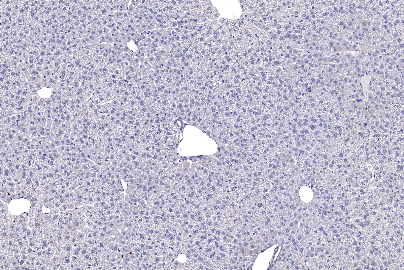

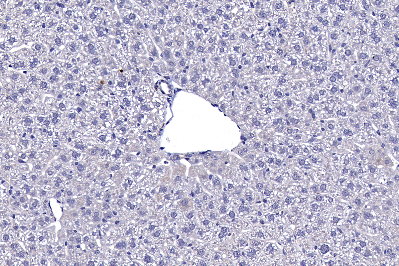
**

*Irg1^f/f^* DEN 48h-Ly6G

**
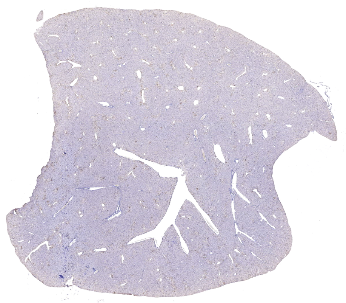

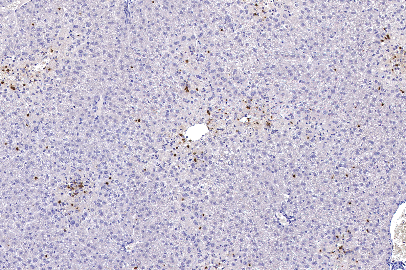

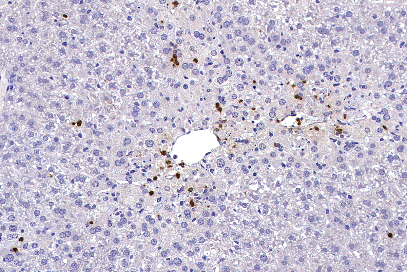
**

*Irg1^hep-/-^* DEN 48h-Ly6G

**
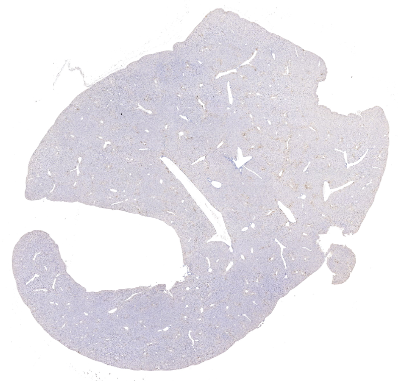

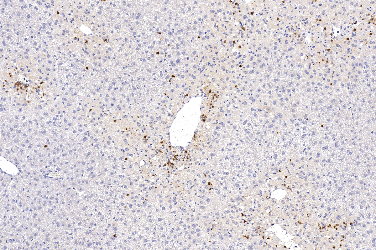

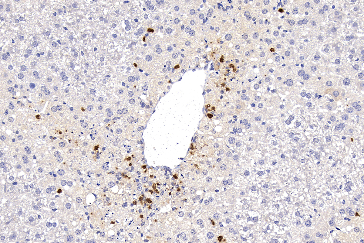
**

**Figure S2B**

Quantification of Ly6G positive cells per high magnification field

| Control | | DEN 48h | |
| --- | --- | --- | --- |
| *Irg1^f/f^* | *Irg1^hep-/-^* | *Irg1^f/f^* | *Irg1^hep-/-^* |
| 0.25 | 0.20 | 32.70 | 30.10 |
| 0.20 | 0.10 | 30.40 | 31.30 |
| 0.10 | 0.40 | 47.80 | 44.20 |
| 0.30 | 0.25 | 34.20 | 36.80 |

*P* = 0. 898 (▲)

**Figure S2C**

*Irg1^f/f^* DEN 0h-F4/80

**
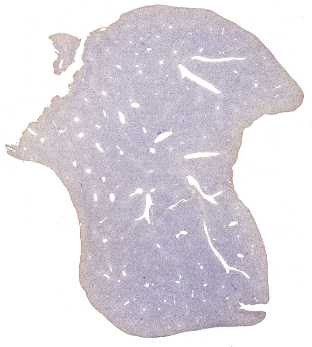

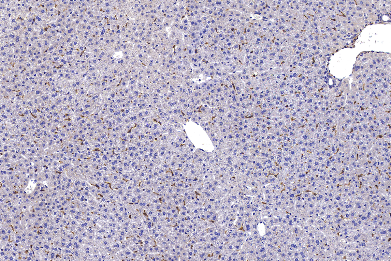

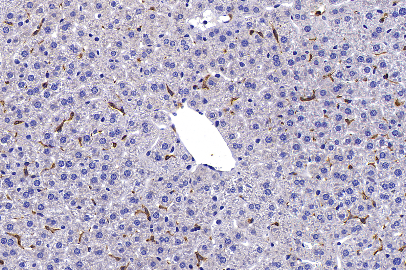
**

*Irg1^hep-/-^* DEN 0h-F4/80


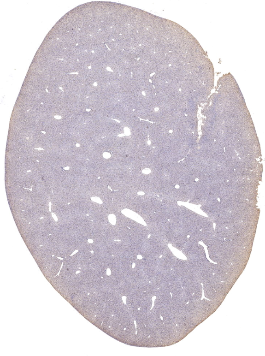

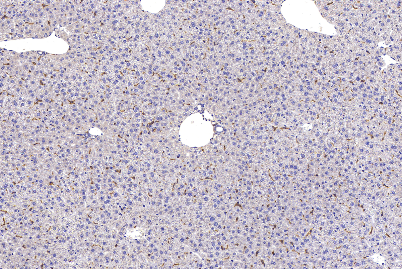

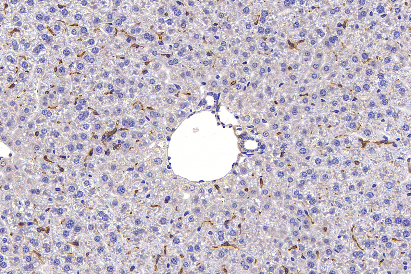


*Irg1^f/f^* DEN 48h-F4/80

**
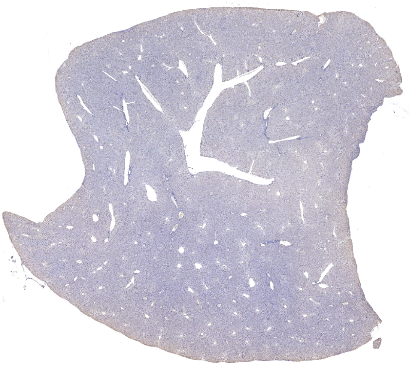

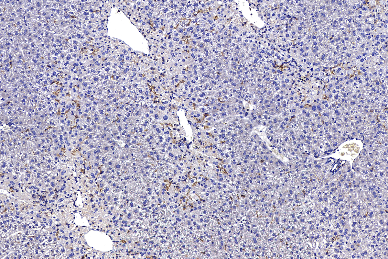

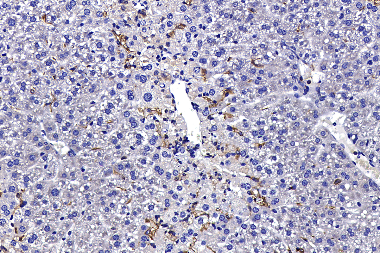
**

*Irg1^hep-/-^* DEN 48h-F4/80

**
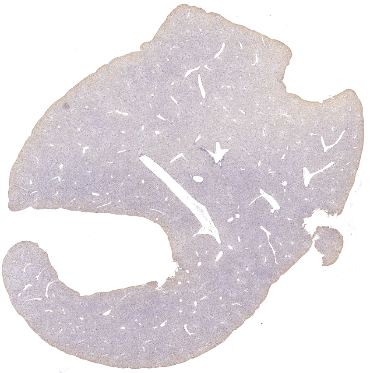

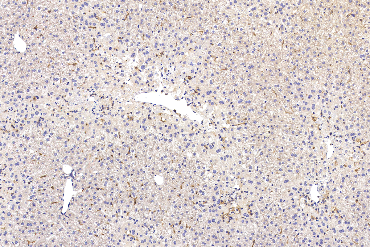

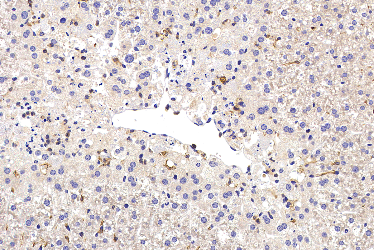
**

**Figure S2D**

Quantification of F4/80 positive cells per high magnification field

| Control | | DEN 48h | |
| --- | --- | --- | --- |
| *Irg1^f/f^* | *Irg1^hep-/-^* | *Irg1^f/f^* | *Irg1^hep-/-^* |
| 26.00 | 25.20 | 22.80 | 21.70 |
| 31.30 | 21.40 | 20.00 | 24.80 |
| 22.90 | 30.00 | 24.10 | 32.30 |
| 32.40 | 33.00 | 26.70 | 20.90 |

*P* = 0.623 (▲)

**Figure S2E**

*Irg1^f/f^* DEN 0h-Ki67

**
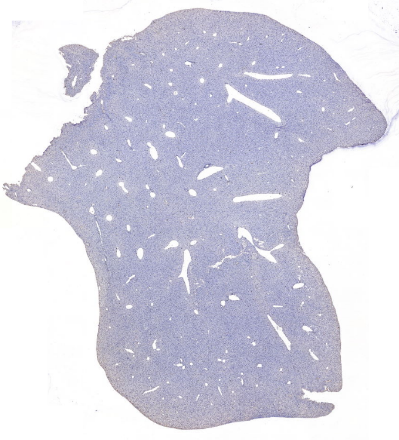

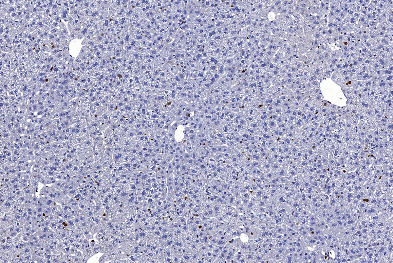

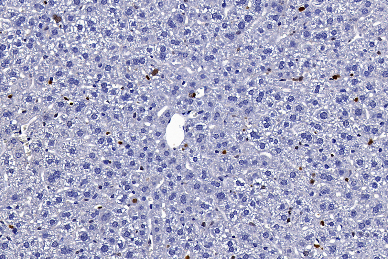
**

*Irg1^hep-/-^* DEN 0h-Ki67


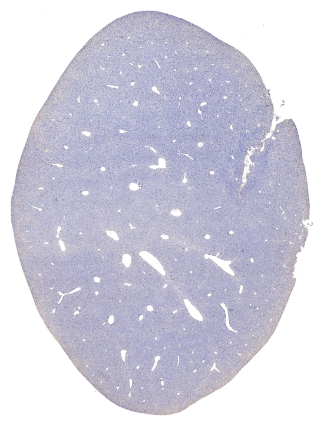

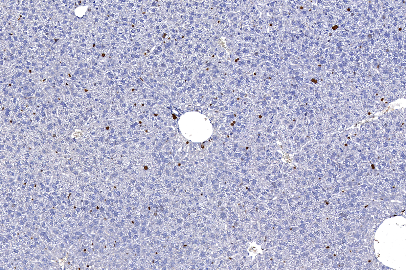

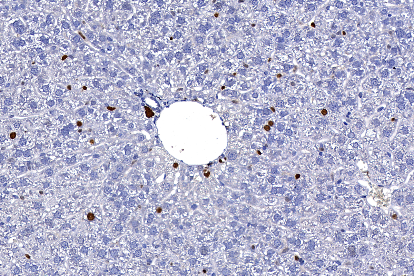


*Irg1^f/f^* DEN 48h-Ki67

**
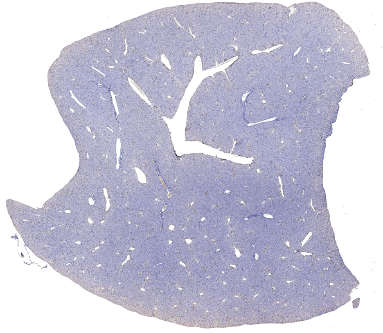

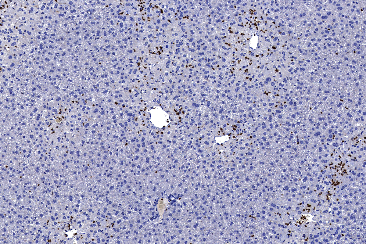

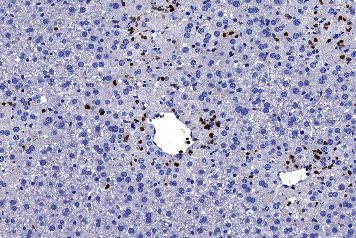
**

*Irg1^hep-/-^* DEN 48h-Ki67


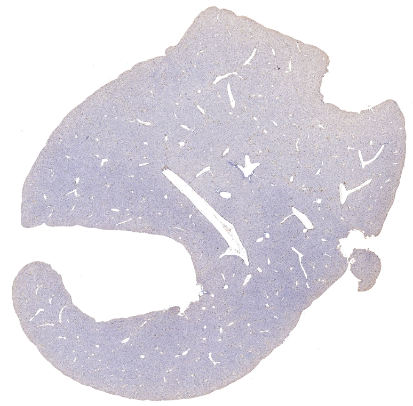

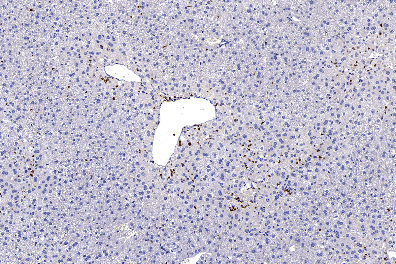


**Figure S2F**

Quantification of Ki67 positive cells per high magnification field

| Control | | DEN 48h | |
| --- | --- | --- | --- |
| *Irg1^f/f^* | *Irg1^hep-/-^* | *Irg1^f/f^* | *Irg1^hep-/-^* |
| 5.00 | 6.90 | 11.00 | 9.00 |
| 4.60 | 5.00 | 7.40 | 7.00 |
| 6.10 | 7.80 | 6.60 | 10.70 |
| 7.80 | 4.00 | 9.00 | 9.40 |

*P* = 0. 686 (▲)

**Figure S2G**

Ctrl

*Irg1^f/f^*

*Irg1^hep-/-^*

DEN 24h Ki67

*Irg1^f/f^*

*Irg1^hep-/-^*

DEN 48h Ki67

*Irg1^f/f^*

*Irg1^hep-/-^*

DEN 72h Ki67

*Irg1^f/f^*

*Irg1^hep-/-^*

**Figure S2H**

| DEN 0h | | DEN 24h | | DEN 48h | | DEN 72h | |
| --- | --- | --- | --- | --- | --- | --- | --- |
| *Irg1^f/f^* | *Irg1^hep-/-^* | *Irg1^f/f^* | *Irg1^hep-/-^* | *Irg1^f/f^* | *Irg1^hep-/-^* | *Irg1^f/f^* | *Irg1^hep-/-^* |
| 20.60 | 21.00 | 16.00 | 15.50 | 28.10 | 31.00 | 9.90 | 12.10 |
| 22.20 | 23.30 | 20.20 | 16.10 | 27.50 | 28.00 | 10.60 | 7.40 |
| 24.00 | 20.20 | 18.90 | 21.00 | 26.90 | 25.40 | 8.40 | 10.20 |
| 20.20 | 22.10 | 17.00 | 18.60 | 30.00 | 25.80 | 7.50 | 9.00 |

DEN 24h *P* = 0.891 (▲), DEN 48h *P* = 0.705 (▲), DEN 72h *P* = 0.653 (▲)

**Figure 3A**

Serum ALT (U/L)

| Control | | APAP 24h | |
| --- | --- | --- | --- |
| *Irg1^f/f^* | *Irg1^hep-/-^* | *Irg1^f/f^* | *Irg1^hep-/-^* |
| 72.6 | 92.8 | 2348.2 | 1258.2 |
| 90.7 | 84.1 | 2097.1 | 1100.4 |
| 75.8 | 87.9 | 2403.7 | 1492.3 |
| 84.4 | 76.3 | 2295.3 | 1168.2 |

*P* < 0.001 (**)

Serum AST (U/L)

| Control | | APAP 24h | |
| --- | --- | --- | --- |
| *Irg1^f/f^* | *Irg1^hep-/-^* | *Irg1^f/f^* | *Irg1^hep-/-^* |
| 179.6 | 140.8 | 2608.6 | 1501.7 |
| 168.2 | 152.9 | 2289.4 | 1165.2 |
| 153.9 | 166.6 | 2578.5 | 1290.6 |
| 162.9 | 157.9 | 2490.2 | 1406.4 |

*P* < 0.001 (**)

**Figure 3B**

*Irg1^f/f^* Control-HE

*Irg1^hep-/-^* Control-HE

*Irg1^f/f^* APAP 24h-HE

*Irg1^hep-/-^* APAP 24h-HE

**Figure 3C**

*Irg1^f/f^* Control-TUNEL

*Irg1^hep-/-^* Control-TUNEL

*Irg1^f/f^* 24h-TUNEL

*Irg1^hep-/-^* 24h-TUNEL

**Figure 3D**

| Control | | APAP 24h | |
| --- | --- | --- | --- |
| *Irg1^f/f^* | *Irg1^hep-/-^* | *Irg1^f/f^* | *Irg1^hep-/-^* |
| 6.80 | 6.00 | 67.00 | 30.5 |
| 5.60 | 4.00 | 72.00 | 23.50 |
| 3.50 | 3.20 | 78.90 | 36.90 |
| 4.00 | 5.80 | 57.20 | 30.00 |

*P* < 0.001 (**)

**Figure 3E**

*Irg1^f/f^* Control-cleaved caspase 3

*Irg1^hep-/-^* Control-cleaved caspase 3

*Irg1^f/f^* APAP 24h-cleaved caspase 3

*Irg1^hep-/-^* APAP 24h-cleaved caspase 3

**Figure 3F**

| Control | | APAP 24h | |
| --- | --- | --- | --- |
| *Irg1^f/f^* | *Irg1^hep-/-^* | *Irg1^f/f^* | *Irg1^hep-/-^* |
| 0.4 | 0 | 28.0 | 8.0 |
| 0 | 0.2 | 26.4 | 10.2 |
| 0 | 0.2 | 28.0 | 12.0 |
| 0 | 0 | 25.6 | 9.8 |

*P* < 0. 001(**)

**Figure 3G**

caspase-3

cleaved caspase-3

cleaved caspase-7

β-actin

**Figure 3H**

IRG1

caspase-3

cleaved caspase-3

cleaved caspase-7

β-actin

**Figure 3I**

IRG1

caspase-3

cleaved caspase-3

cleaved caspase-7

β-actin

**Figure 3J**

BNL CL.2

Flag-B medium

Flag-B APAP 24h

Flag-IRG1 medium

Flag-IRG1 APAP 24h

**Figure 3K**

HHL-5

Flag-B medium

Flag-B APAP 24h

Flag-IRG1 medium

Flag-IRG1 APAP 24h

**Figure S3A**

*Irg1^f/f^* Control-Ly6G

*Irg1^hep-/-^* Control-Ly6G

*Irg1^f/f^* APAP 24h-Ly6G

*Irg1^hep-/-^* APAP 24h-Ly6G

**Figure S3B**

| Control | | APAP 24h | |
| --- | --- | --- | --- |
| *Irg1^f/f^* | *Irg1^hep-/-^* | *Irg1^f/f^* | *Irg1^hep-/-^* |
| 0.10 | 0.40 | 34.20 | 30.8 |
| 0.00 | 0.10 | 29.7 | 25.9 |
| 0.30 | 0.00 | 30.9 | 36.9 |
| 0.00 | 0.10 | 32.8 | 27.0 |

*P* = 0.537 (▲)

**Figure S3C**

*Irg1^f/f^* Control-F4/80

*Irg1^hep-/-^* Control-F4/80

*Irg1^f/f^* APAP 24h-F4/80

*Irg1^hep-/-^* APAP 24h-F4/80

**Figure S3D**

| Control | | APAP 24h | |
| --- | --- | --- | --- |
| *Irg1^f/f^* | *Irg1^hep-/-^* | *Irg1^f/f^* | *Irg1^hep-/-^* |
| 30.20 | 32.50 | 24.6 | 29.8 |
| 36.80 | 30.90 | 28.4 | 30.0 |
| 32.60 | 26.0 | 30.0 | 22.4 |
| 28.90 | 27.8 | 23.7 | 25.9 |

*P* = 0.887 (▲)

**Figure S3E**

| Groups | Proportion of Annexin V positive cells (%) |
| --- | --- |
| Empty vector | 2.2 |
|  | 3.5 |
|  | 3.1 |
|  | 2.9 |
|  |  |
| Empty vector + APAP | 7.0 |
|  | 6.9 |
|  | 6.2 |
|  | 6.4 |
|  |  |
| Empty vector + APAP + Z-VAD-FMK | 4.1 |
|  | 3.9 |
|  | 3.8 |
|  | 4.7 |
|  |  |
| IRG1 | 8.2 |
|  | 9.1 |
|  | 10.8 |
|  | 8.1 |
|  |  |
| IRG1+APAP | 17.1 |
|  | 18.9 |
|  | 19.3 |
|  | 18.4 |
|  |  |
| IRG1 + APAP + Z-VAD-FMK | 4.7 |
|  | 5.0 |
|  | 4.2 |
|  | 5.2 |

**Figure S3F**

| Groups | Proportion of Annexin Ⅴ positive cells (%) |
| --- | --- |
| Empty vector | 6.4 |
|  | 5.7 |
|  | 6.3 |
|  | 6.6 |
|  |  |
| Empty vector + APAP | 17.6 |
|  | 14.4 |
|  | 18.0 |
|  | 15.3 |
|  |  |
| Empty vector + APAP + Z-VAD-FMK | 8.3 |
|  | 7.9 |
|  | 8.4 |
|  | 7.2 |
|  |  |
| IRG1 | 12.7 |
|  | 11.5 |
|  | 12.0 |
|  | 11.3 |
|  |  |
| IRG1+APAP | 26.6 |
|  | 28.9 |
|  | 24.4 |
|  | 25.9 |
|  |  |
| IRG1+APAP+Z-VAD-FMK | 9.0 |
|  | 7.8 |
|  | 8.4 |
|  | 8.2 |

**Figure S3G**

| Empty vector | 1.0 |
| --- | --- |
|  | 2.3 |
|  | 1.7 |
|  | 1.3 |
|  |  |
| Empty vector + APAP | 5.4 |
|  | 6.3 |
|  | 7.5 |
|  | 4.6 |
|  |  |
| IRG1 | 6.0 |
|  | 6.9 |
|  | 4.8 |
|  | 5.3 |
|  |  |
| IRG1 + APAP | 12.6 |
|  | 10.2 |
|  | 14.4 |
|  | 12.2 |

**Figure S3H**

| Empty vector | 2.4 |
| --- | --- |
|  | 2.5 |
|  | 1.9 |
|  | 2.3 |
|  |  |
| Empty vector + APAP | 7.0 |
|  | 9.6 |
|  | 6.3 |
|  | 6.9 |
|  |  |
| IRG1 | 5.4 |
|  | 6.7 |
|  | 5.5 |
|  | 6.3 |
|  |  |
| IRG1 + APAP | 10.3 |
|  | 12.6 |
|  | 10.8 |
|  | 11.4 |

**Figure 4A**

Serum ALT (U/L)

| Control + DEN 0h | 4-OI + DEN 0h | Control + DEN 48h | 4-OI + DEN 48h |
| --- | --- | --- | --- |
| 76.1 | 74.6 | 1370.2 | 829.7 |
| 82.9 | 79.3 | 1180.5 | 1200.5 |
| 79.7 | 84.5 | 921.8 | 1004.2 |
| 86.4 | 87.9 | 1207.6 | 1190.4 |

*P* = 0.407 (▲)

Serum AST (U/L)

| Control + DEN 0h | 4-OI + DEN 0h | Control + DEN 48h | 4-OI + DEN 48h |
| --- | --- | --- | --- |
| 140.2 | 138.5 | 1398.4 | 1000.6 |
| 152.5 | 145.7 | 1023.7 | 1420.8 |
| 146.9 | 149.4 | 1200.5 | 1307.5 |
| 151.8 | 143.8 | 1187.3 | 1038.7 |

*P* = 0.937 (▲)

**Figure 4B**

Control+DEN 0h-HE

4OI+DEN 0h-HE

Control + DEN 24h-HE

4-OI + DEN 24h-HE

Control + DEN 48h-HE

4-OI + DEN 48h-HE

**Figure 4C**

Control + DEN 0h-TUNEL

4-OI + DEN 0h-TUNEL

Control + DEN 48h-TUNEL

4-OI + DEN 48h-TUNEL

**Figure 4D**

| Control + DEN 0h | 4-OI + DEN 0h | Control + DEN 48h | 4-OI + DEN 48h |
| --- | --- | --- | --- |
| 3.8 | 4.5 | 26 | 27.3 |
| 4.7 | 4.0 | 32.4 | 31.1 |
| 6.1 | 3.2 | 37.2 | 26.6 |
| 3.9 | 5.8 | 27.5 | 30.8 |

*P* = 0.538 (▲)

**Figure 4E**

caspase-3

cleaved caspase-3

cleaved caspase-7

β-actin

**Figure 4F**

Serum ALT (U/L)

| Control + APAP 0h | 4-OI + APAP 0h | Control + APAP 24h | 4-OI + APAP 24h |
| --- | --- | --- | --- |
| 82.0 | 79.7 | 2034.8 | 2400.8 |
| 74.9 | 74.3 | 2207.1 | 1896.3 |
| 72.0 | 80.5 | 1908.2 | 2139.5 |
| 76.2 | 72.4 | 1907.4 | 2069.6 |

*P* = 0.409 (▲)

Serum AST (U/L)

| Control + APAP 0h | 4-OI + APAP 0h | Control + APAP 24h | 4-OI + APAP 24h |
| --- | --- | --- | --- |
| 156.8 | 140.4 | 2505.3 | 2386.1 |
| 142.8 | 149.9 | 2013.9 | 2170.8 |
| 148.9 | 146.5 | 2417.7 | 2703.6 |
| 140.2 | 141.6 | 2107.5 | 2248.9 |

*P* = 0.512 (▲)

**Figure 4G**

Control + APAP 0h-HE

4-OI + APAP 0h-HE

Control + APAP 24h-HE

4-OI + APAP 24h-HE

**Figure 4H**

Control + APAP 0h-TUNEL

4-OI + APAP 0h-TUNEL

Control + APAP 24h-TUNEL

4-OI + APAP 24h-TUNEL

**Figure 4I**

| Control + APAP 0h | 4-OI + APAP 0h | Control + APAP 24h | 4-OI + APAP 24h |
| --- | --- | --- | --- |
| 3.00 | 3.40 | 41.55 | 44.91 |
| 3.60 | 2.60 | 42.18 | 35.27 |
| 2.40 | 2.90 | 37.87 | 40.68 |
| 2.50 | 3.80 | 45.90 | 41.60 |

*P* = 0.644 (▲)

**Figure 4J**

caspase-3

cleaved caspase-3

cleaved caspase-7

β-actin

**Figure S4A**

Control + DEN 0h-cleaved caspase-3

4-OI + DEN 0h-cleaved caspase-3

Control + DEN 48h-cleaved caspase-3

4-OI + DEN 48h-cleaved caspase-3

**Figure S4B**

| Control + DEN 0h | 4-OI + DEN 0h | Control + DEN 48h | 4-OI + DEN 48h |
| --- | --- | --- | --- |
| 0.3 | 0.3 | 43.4 | 34.1 |
| 0.9 | 0.5 | 34.5 | 30.2 |
| 0.4 | 0.6 | 32.9 | 40.8 |
| 0.4 | 0.2 | 40.2 | 32.9 |

*P* = 0.367 (▲)

**Figure S4C**

Control + APAP 0h-cleaved caspase-3

4-OI + APAP 0h-cleaved caspase-3

Control + APAP 24h-cleaved caspase 3

4-OI + APAP 24h-cleaved caspase-3

**Figure S4D**

| Control + APAP 0h | 4-OI + APAP 0h | Control + APAP 24h | 4-OI + APAP 24h |
| --- | --- | --- | --- |
| 0.20 | 0.30 | 41.98 | 31.55 |
| 0.30 | 0.30 | 37.79 | 32.09 |
| 0.20 | 0.20 | 30.14 | 37.92 |
| 0.20 | 0.20 | 34.96 | 40.89 |

*P* = 0.863 (▲)

**Figure S4E**

BNL CL.2

caspase-3

cleaved caspase-3

cleaved caspase-7

β-actin

**Figure S4F**

HHL-5

caspase-3

cleaved caspase-3

cleaved caspase-7

β-actin

**Figure S4G**

DMSO APAP 0h

DMSO APAP 24h

4-OI APAP 0h

4-OI APAP 24h

**Figure S4H**

DMSO APAP 0h

DMSO APAP 24h

4-OI APAP 0h

4-OI APAP 24h

**Figure 5A**

Tomm-20

Flag-IRG1

DAPI

Merge

**Figure 5B**

cytochrome *c*

Bax

GAPDH

**Figure 5C**

cytochrome *c*

Bax

GAPDH

**Figure 5D**

Bax

cytochrome *c*

SDHA

**Figure 5E**

Bax

cytochrome *c*

SDHA

**Figure 5F**

cytochrome *c*

Bax

GAPDH

**Figure 5G**

cytochrome *c*

Bax

GAPDH

**Figure 5H**

Bax

cytochrome *c*

SDHA

**Figure 5I**

Bax

cytochrome *c*

SDHA

**Figure S5A**

caspase-8

cleaved caspase-8

β-actin

**Figure S5B**

caspase-8

cleaved caspase-8

β-actin

**Figure S5C**

caspase 8

cleaved caspase 8

β-actin

**Figure S5D**

caspase 8

cleaved caspase 8

β-actin

**Figure 6A**

IB Mcl-1

IB IRG1

IRG1 input

MCL-1 input

**Figure 6B**

IB IRG1

IB Mcl-1

IRG1 input

MCL-1 input

**Figure 6C**

IB Mcl-1

IB IRG1

IRG1 input

MCL-1 input

**Figure 6D**

IB IRG1

IB Mcl-1

IRG1 input

MCL-1 input

**Figure 6E**

IB Bim

IB Mcl-1

Bim input

MCL-1 input

**Figure 6F**

IB Bim

IB Mcl-1

Bim input

MCL-1 input

**Figure 6G**

IB Mcl-1

IB Bim

Bim input

MCL-1 input

**Figure 6H**

IB Mcl-1

IB Bim

Bim input

MCL-1 input

**Figure 6I**

IB Bim

IB Mcl-1

Bim input

MCL-1 input

**Figure 6J**

IB Bim

IB Mcl-1

Bim input

MCL-1 input

**Figure 6K**

IB Mcl-1

IB Bim

Bim input

MCL-1 input

**Figure 6L**

IB Mcl-1

IB Bim

Bim input

MCL-1 input

**Figure 6M**

IB V5 (left)

IB Flag (left)

IB V5 (right)

IB Flag (right)

**Figure 6N**

IB V5

IB Flag

**Figure 6O**

IP Flag IB V5

IB Flag

**Figure S6A**

**Figure S6B**

**Figure S6C**

Mcl-1

β-actin

**Figure S6D**

Bax

SDHA

**Figure S6E**

cytochrome *c*

GAPDH

**Figure S6F**

Bim

β-actin

**Figure S6G**

Bax

SDHA

**Figure S6H**

cytochrome *c*

GAPDH

**Figure S6I**

IB V5

IB Flag

V5 input

Flag input

**Figure S6J**

IB V5

IB Flag

V5 input

Flag input

**Figure 7**
